# Formation of novel transition metal hydride complexes with ninefold hydrogen coordination

**DOI:** 10.1038/srep44253

**Published:** 2017-03-13

**Authors:** Shigeyuki Takagi, Yuki Iijima, Toyoto Sato, Hiroyuki Saitoh, Kazutaka Ikeda, Toshiya Otomo, Kazutoshi Miwa, Tamio Ikeshoji, Shin-ichi Orimo

**Affiliations:** 1 Institute for Materials Research, Tohoku University, Sendai 980-8577, Japan; 2 Quantum Beam Science Research Directorate, National Institute for Quantum and Radiological Science and Technology, Hyogo 679-5148, Japan; 3 Institute of Materials Structure Science, High Energy Accelerator Research Organization, Tsukuba 305-0801, Japan; 4 Toyota Central R&D Laboratories, Inc., Nagakute 480-1192, Japan; 5 WPI-Advanced Institute for Materials Research, Tohoku University, Sendai 980-8577, Japan

## Abstract

Ninefold coordination of hydrogen is very rare, and has been observed in two different hydride complexes comprising rhenium and technetium. Herein, based on a theoretical/experimental approach, we present evidence for the formation of ninefold H- coordination hydride complexes of molybdenum ([MoH_9_]^3−^), tungsten ([WH_9_]^3−^), niobium ([NbH_9_]^4−^) and tantalum ([TaH_9_]^4−^) in novel complex transition-metal hydrides, Li_5_MoH_11_, Li_5_WH_11_, Li_6_NbH_11_ and Li_6_TaH_11_, respectively. All of the synthesized materials are insulated with band gaps of approximately 4 eV, but contain a sufficient amount of hydrogen to cause the H 1*s*-derived states to reach the Fermi level. Such hydrogen-rich materials might be of interest for high-critical-temperature superconductivity if the gaps close under compression. Furthermore, the hydride complexes exhibit significant rotational motions associated with anharmonic librations at room temperature, which are often discussed in relation to the translational diffusion of cations in alkali-metal dodecahydro-*closo*-dodecaborates and strongly point to the emergence of a fast lithium conduction even at room temperature.

Although exhibiting the simplest form, hydrogen has an exceptionally rich chemistry, forming various chemical bonds in materials^1^. The flexibility of hydrogen is the source of many interesting functionalities in hydrides, such as hydrogen storage^2^, superconductivity^3^,^4^,^5^,^6^, fast ionic conductivity^7^, magnetism^8^ and metal–insulator transition^9^,^10^; thus, materials containing a large amount of hydrogen are promising candidates not only for improving material performance but also for the emergence of latent functionalities.

Complex transition metal hydrides represent a class of hydrogen-rich materials, wherein a number of H atoms covalently bind to a transition metal element (*T*) to form hydride complexes with a remarkably rich variety of H-coordination modes ranging from twofold to ninefold^1^,^11^,^12^,^13^,^14^,^15^,^16^,^17^,^18^,^19^,^20^,^21^,^22^,^23^,^24^,^25^. These complexes are stabilized by charge transfer from electropositive counterions, such as alkali and alkaline earth metals, to form insulating hydrides^1^,^20^,^23^. In general, the H-coordination number increases from right to left along a given period because of the trend of increasing atomic size, which allows more hydrogen binding. At the same time, despite research spanning the past several decades, the elements capable of forming hydride complexes are thought to be limited to those in Groups 7–12^18^. This limitation has so far precluded further discovery of hydride complexes with high H-coordination. For example, the presently known members with the ninefold H-coordination are limited to those comprising the Group 7 elements technetium ([TcH_9_]^2−^)^11^ and rhenium ([ReH_9_]^2−^)^12^,^13^,^14^,^16^,^17^. However, we recently reported the formation of the first Group 6 hydride complex [CrH_7_]^5−^ in Mg_3_CrH_8_^25^, thereby demonstrating that there is no *a priori* reason for this limitation and paving the way for the discovery of higher H-coordination hydride complexes comprising unexplored elements to the left of Group 7.

To demonstrate the feasibility of this strategy, we have experimentally examined the formation of novel transition metal hydride complexes of Group 5 and Group 6 elements (Mo, W, Nb and Ta) based on a first-principles prediction. Lithium was selected as a counterion because of its low electronegativity, which is expected to thermodynamically stabilize the complex hydride phases^1^,^20^,^23^. The crystal structures and electronic structures of the obtained samples were investigated using a combination of experimental and *ab initio* approaches. Finally, we discuss the prospects for the emergence of H-driven functionalities in the obtained materials.

## Results

### First-principles predictions

**Figure 1:**
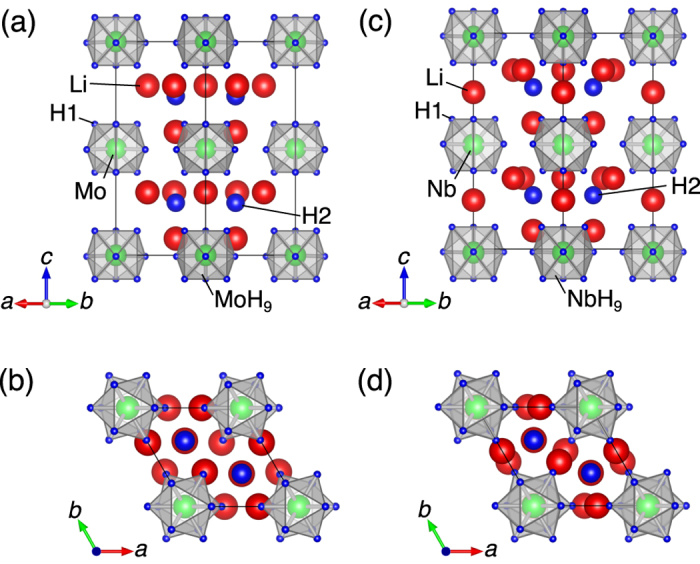
Crystal structures. (**a**–**d**) Density functional theory (DFT) structures of Li_5_MoH_11_ (**a**,**b**) and Li_6_NbH_11_ (**c,d**) viewed along the [110] (**a,c**) and [001] (**b,d**) directions. Li_5_WH_11_ and Li_6_TaH_11_ have isostructures to Li_5_MoH_11_ and Li_6_NbH_11_, respectively.

We first performed a ground-state structure search of Li–Mo–H and Li–Nb–H systems using first-principles calculations. Figure 1 shows the obtained most stable structures found with stoichiometries of Li_5_MoH_11_ and Li_6_NbH_11_. In Li_5_MoH_11_, nine H atoms (H1) form a tricapped trigonal prism around the Mo atoms sitting at the (0, 0, 0) and (0, 0, 1/2) coordinates of the hexagonal lattice and two isolated H atoms (H2) are located at the approximate centres of the trigonal prisms formed by the six Mo atoms. The four Li atoms coordinate to the H2 atoms, forming a two-dimensional network of corner-sharing tetrahedra between the layers of MoH_9_ units. Li_6_NbH_11_ has a structure similar to that of Li_5_MoH_11_; however, it has an additional Li site between the NbH_9_ tricapped trigonal prisms along the *c*-axis, which accommodates the 6:1 stoichiometry between Li and Nb.

The calculated standard heats of formation 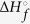 are −510 kJ/mol for Li_5_MoH_11_ and −637 kJ/mol for Li_6_NbH_11_, which are much lower than those of the conceivable decomposed products, 5LiH + Mo + 3H_2_ and 6LiH + NbH_0.65_ + 2.175H_2_, respectively; therefore, the materials could potentially be synthesized via the following reactions: 5LiH + Mo + 3H_2_ → Li_5_MoH_11_ and 6LiH + NbH_0.65_ + 2.175H_2_ → Li_5_MoH_11_, with the enthalpies of reaction Δ*H*_r_ of −105 and −91 kJ/mol, respectively (note that we used −81 kJ/mol^26^ for LiH and −60 kJ/mol for NbH_0.65_^27^). We observed that the isostructural 5*d* analogues Li_5_WH_11_ and Li_6_TaH_11_ also have higher thermodynamic stabilities of −529 and −651 kJ/mol with respect to the conceivable decomposition products 5LiH + W + 3H_2_ and 6LiH + TaH_0.65_ + 2.175H_0.65_, respectively (note that the enthalpies of reaction are estimated to be −128 and −117 kJ/mol, respectively, using −51 kJ/mol for TaH_0.65_^28^). The results are summarized in Table 1.

### Electronic structures

**Figure 2:**
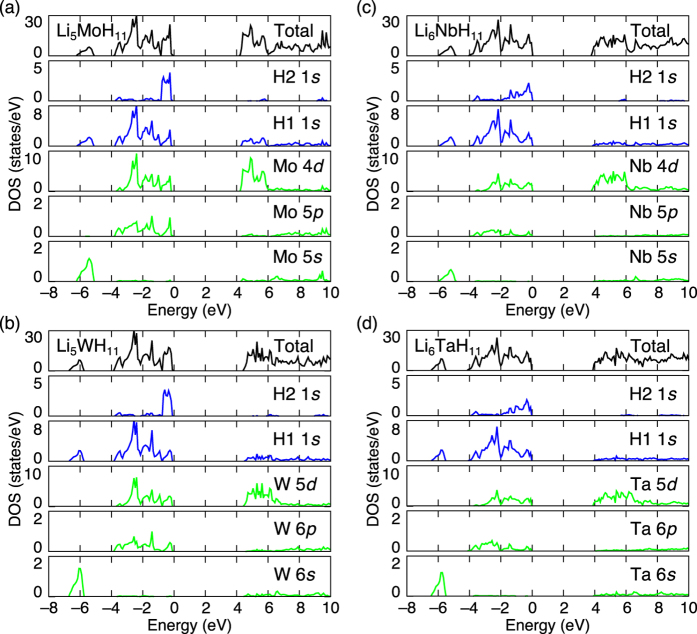
Electronic structures. Total electronic density of states (DOS; top panels) and H2 1*s* (second panel from the top), H1 1*s* (third panel from the top), *T d* (third panel from the bottom), *T p* (second panel from the bottom) and *T s* (bottom panels) projections for Li_5_MoH_11_ (**a**), Li_5_WH_11_ (**b**), Li_6_NbH_11_ (**c**) and Li_6_TaH_11_ (**d**). The energy zero is set at the valence-band maximum.

Based on the obtained structures, we discuss the electronic structures. Figure 2 shows the calculated electronic densities of states (DOSs) and the H *s* and *T spd* projections. We checked for magnetic states and observed no magnetic ordering in any of the materials.

The most important feature of the electronic structures is that the 1*s* states of the nine H1 atoms forming the *T*H_9_ tricapped trigonal prisms reach the Fermi level (*E*_F_) in all materials, as observed in the third panels from the top in Fig. 2a–d. These states strongly hybridize with the *T spd* states to form the *σ*-bonds, which is evident from the H1 *s* and *T spd* characters of both the valence and conduction bands. These results are consistent with a previous electronic structure calculation by Singh *et al*.^29^ for BaReH_9_, which contains the hydride complex ReH_9_. Because all nine bonding states are fully occupied by 18 electrons per formula unit, the *T*H_9_ units can be considered as the hydride complexes [MoH_9_]^3−^, [WH_9_]^3−^, [NbH_9_]^4−^ and [TaH_9_]^4−^. The 1*s* states of the isolated H2 atoms lie immediately below *E*_F_ without noticeable hybridization with any other states, as observed in the second panels from the top in Fig. 2a–d. Thus, these atoms can be considered as H^−^ ions. The electropositive Li states occur far above *E*_F_ (not shown) and donate one electron per atom to the hydride complexes to become Li^+^ cations. Thus, we conclude that the ionic configurations of the current materials are [Li^+^]_5_[MoH_9_]^3−^[H^−^]_2_, [Li^+^]_5_[WH_9_]^3−^[H^−^]_2_, [Li^+^]_6_[NbH_9_]^4−^[H^−^]_2_ and [Li^+^]_6_[TaH_9_]^4−^[H^−^]_2_. The band gaps are approximately 4 eV in all of the materials.

### Syntheses

We experimentally examined the possibility of the formation the theoretically predicted complex transition metal hydrides using a high-pressure and high-temperature technique. Powdered mixtures of LiH and Group 5 and Group 6 transition metals were hydrogenated under 5 GPa at 923–1023 K for 24–48 h. The colours of the samples recovered at ambient pressure and temperature were yellow for Li–Mo–H, greenish yellow for Li–W–H, light grey for Li–Nb–H and white for Li–Ta–H, implying the insulating character, as is typical with complex transition-metal hydrides.

**Figure 3:**
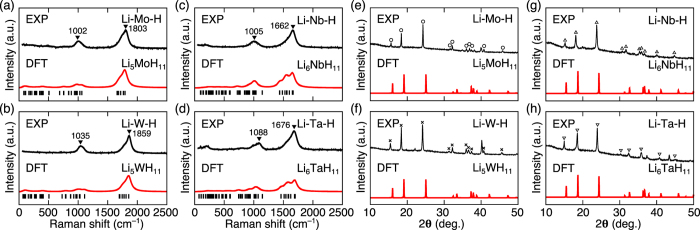
Raman spectra and X-ray diffraction (XRD) profiles. (**a**–**d**) Raman spectra. Experimental spectra (black lines) and DFT spectra (red lines) for Li_5_MoH_11_ (**a**), Li_5_WH_11_ (**b**), Li_6_NbH_11_ (**c**) and Li_6_TaH_11_ (**d**). All of the zone-centre optical phonon frequencies obtained from DFT calculations are represented by tick marks for reference. (**e**–**h**) XRD profiles. Experimental profiles (black lines) and simulated profiles from DFT structures (red lines) for Li_5_MoH_11_ (**e**), Li_5_WH_11_ (**f**), Li_6_NbH_11_ (**g**) and Li_6_TaH_11_ (**h**). The open circles, cross marks, open triangles and open upside–down triangles indicate the diffraction peaks correspond to Li_5_MoH_11_, Li_5_WH_11_, Li_6_NbH_11_ and Li_6_TaH_11_, respectively.

The recovered samples were characterized by Raman spectroscopy, and the results are illustrated in Fig. 3a–d (black lines) along with the density functional theory (DFT) spectra (red lines) calculated using a method reported in the literature^30^. Because the primitive cells of Li_5_MoH_11_/Li_5_WH_11_ and Li_6_NbH_11_/Li_6_TaH_11_ comprise 34 and 36 atoms, respectively, there are a total of 99 (10*A*_1_ + 6*A*_2_ + 6*B*_1_ + 11*B*_2_ + 17*E*_2_ + 16*E*_1_) and 105 (11*A*_1_ + 6*A*_2_ + 6*B*_1_ + 12*B*_2_ + 18*E*_2_ + 17*E*_1_) zone-centre optical phonon modes, respectively, as represented by the tick marks in Fig. 3a–d. Among them, 76 modes (10*A*_1_ + 17*E*_2_ + 16*E*_1_) of Li_5_MoH_11_/Li_5_WH_11_ and 81 modes (11*A*_1_ + 18*E*_2_ + 17*E*_1_) of Li_6_NbH_11_/Li_6_TaH_11_ are Raman active. These modes provide an intense and asymmetric band at approximately 1660–1860 cm^−1^ and a weak band at approximately 1000 cm^−1^ in all the DFT spectra, which originate from the *T*–H stretching and H–*T*–H bending vibrations, respectively. The experimental spectra exhibit very similar features to those obtained by the DFT calculations, strongly supporting the formation of the theoretically predicted ninefold H-coordination hydride complexes.

Figure 3e–h shows X-ray diffraction (XRD) profiles of the recovered samples (black lines) and the profiles simulated based on the DFT structures (red lines). Although the samples contain small amounts of impurities, the Bragg peaks were well indexed to a similar hexagonal lattice with the lattice parameters listed in Table 2. While all of the Bragg peaks are considered to primarily originate from diffraction by the transition metals due to the small atomic scattering factors of H and Li, we successfully reproduced the profiles by placing transition metals at the (0, 0, 0) and (0, 0, 1/2) coordinates, which is similar to the DFT structures. The low concentrations of transition metals suggest the existence of large amounts of Li and H atoms along with transition metals.

### Finite-temperature effects

The DFT lattice parameters summarized in Table 2 are slightly smaller than those determined from the XRD profiles measured at room temperature (4–10% smaller in volume), implying the presence of finite-temperature effects. As such, we examined the dynamics of the room-temperature phase of Li_5_MoH_11_ as an example using both first-principles molecular dynamics (FPMD) calculations in the isothermal–isobaric (*NPT*) ensemble at 298 K and neutron diffraction (ND) measurements at room temperature (strictly, ND measuremts were performed on the deuteride analogue Li_5_MoD_11_).

**Figure 4:**
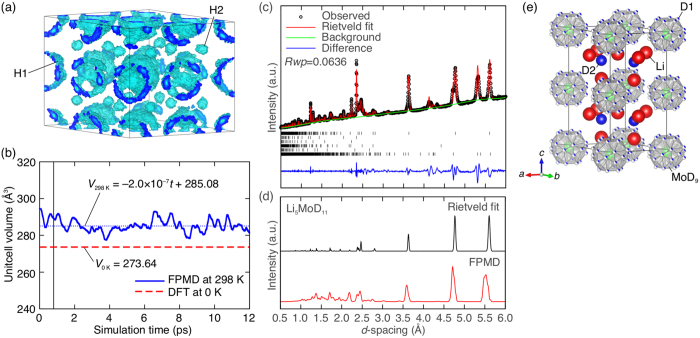
Finite-temperature effects in Li_5_MoH_11_. (**a**) The time-averaged atomic-density profile from the FPMD trajectory of H atoms at 298 K. (**b**) The time-dependent variation of unit cell volume from FPMD calculations at 298 K (blue line). The dashed blue line is obtained by the least-squares fitting of the volume change at 298 K over a range from 0.8 ps (vertical line) to 12 ps. The grand-state unit cell volume obtained by the DFT structure optimization at 0 K is plotted as a reference (dashed red line). (**c**) Experimental ND profile of Li_5_MoD_11_ measured at room temperature. The positions of Bragg reflection (tick marks) are shown for Li_5_MoD_11_ (top), LiD (second top), Mo (third top), LiOD (third bottom), Li_2_O (second bottom) and the unknown phase (bottom). The weight fractions of each phase excluding the unknown phase are Li_5_MoD_11_: 32(1) wt.%, LiD: 13(1) wt.%, Mo: 49(1) wt.%, LiOD: 3 wt.% and Li_2_O: 3 wt.%. The weight fraction of LiOD and Li_2_O are not allowed to refine. (**d**) Rietveld fit of experimental profile (black line) and simulated profile from FPMD trajectory at 298 K (red line). (**e**) Crystal structure of Li_5_MoD_11_ determined by Rietveld analysis.

In FPMD calculations, the two isolated H atoms (H2) isotropically fluctuate around their original positions determined by DFT calculations at 0 K, whereas the nine H atoms in the MoH_9_ units (H1) rotate around the Mo atoms, generating spherical shell-like distribution, as illustrated in Fig. 4a. This type of rotational motion associated with anharmonic librations is acknowledged as leading to phase transitions in borohydrides^31^ and is thus expected to promote the volume expansion at room temperature. In fact, we observed a ~4% expansion (285 Å^3^) relative to the ground-state volume obtained by DFT calculations at 0 K (274 Å^3^), as shown in Fig. 4b. Additionally, the FPMD trajectory reasonably reproduces the experimental ND profile in Fig. 4c, as observed by the comparison of the simulated profile from the trajectory (red line in Fig. 4d) with the Rietveld refinement fit (black line in Fig. 4d), where the *P*6*cc* structure (No. 184) with nine disordered D atoms surrounding the Mo atoms was employed, as shown in Fig. 4e, suggesting the adequacy of the current discussion. The experimentally determined structural parameters are summarized in Table 3.

## Discussion

We have examined the possibility of the formation of novel transition metal hydride complexes with high H-coordination using a combined theoretical/experimental approach. All experimental data strongly point to the successful syntheses of a series of theoretically predicted complex transition metal hydrides Li_5_MoH_11_, Li_5_WH_11_, Li_6_NbH_11_ and Li_6_TaH_11_ containing ninefold H-coordination hydride complexes and two H^−^ anions. Our theoretical calculations reveal the *σ*-bonds of the nine H atoms forming the hydride complexes and the transition metals reach *E*_F_ in all synthesized materials.

**Figure 5:**
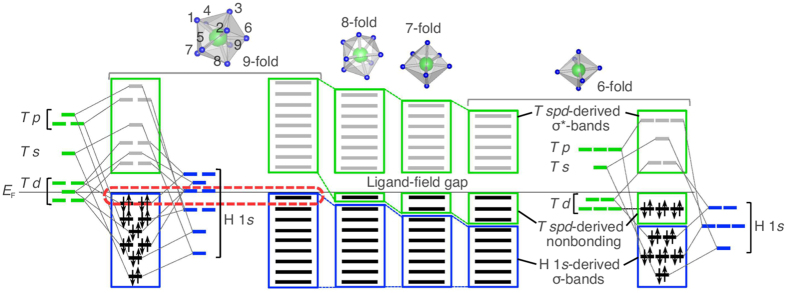
Strategy for obtaining complex transition-metal hydrides with H 1*s*-derived states reaching the Fermi level (*E*_F_). The electronic structures roughly comprise low-lying H 1*s*- and high-lying *T spd*-derived states. The contribution from H 1*s*-derived states to the valence bands increases with increasing H-coordination number and thus reaches *E*_F_ at ninefold H-coordination, as highlighted by the red dashed circle.

Generally, the electronic structures of complex transition-metal hydrides can be viewed as comprising low-lying H 1*s*- and high-lying *T spd*-derived states, as schematically depicted in Fig. 5. The ligand field induced by the H-coordination divides the *T spd* states into the nonbonding and antibonding states (σ^*^-bands), and *E*_F_ falls in the ligand-field gap to accommodate the so-called 18-electron rule. Therefore, the valence bands around *E*_F_ are normally dominated by the *T spd*-derived nonbonding states (strictly, the *T d*-derived nonbonding states); however, we emphasize here that the contribution from the H 1*s*-derived *σ*-bands in the valence bands increases with increasing H-coordination number, pushing the *T spd*-derived states out of the valence bands and reaching *E*_F_ at ninefold coordination (Fig. 5). This is a novel strategy for obtaining solid-state covalently bonded hydrides with the H 1*s*-derived states reaching *E*_F_.

As is well known, it is indispensable to have ‘*σ*-bands’ of light elements at *E*_F_ to achieve high-critical-temperature (high-*T*_c_) superconductivity within the Bardeen–Cooper–Schrieffer (BCS) theory^32^. For example, the relatively high *T*_c_ observed in MgB_2_^33^ is primarily attributed to the high-lying two-dimensional *σ*-bands derived from the in-plane B–B bonds, the holes of which strongly couple with the bond stretch phonons^34^. Conversely, in all of the materials synthesized in this study, nine of the lightest H atoms form strong *σ*-bonds with transition metal elements, providing a DOS just below *E*_F_. In this context, the occurrence of stronger electron–phonon coupling may be expected if the band gaps close under compression. From our preliminary calculations, we observed that there is at least one stable metallic phase of Li_5_MoH_11_ above 94 GPa (see Supplementary information).

Besides the electronic feature, these materials have a characteristic phonon property such that the ninefold hydride complexes exhibit rotational motions associated with anharmonic librations at room temperature. This type of motion has never been observed in the complex transition metal hydrides, but has often been discussed in relation to the occurrence of fast ion conduction in alkali metal dodecahydro-*closo*-dodecaborates such as Na_2_B_12_H_12_. In this material, the first-order phase transition from the low-temperature monoclinic to the high-temperature cubic phase occurs near 520 K, which is accompanied by an increase of two orders of magnitude in the rate of reorientational jumps of the icosahedral [B_12_H_12_]^2−^ anions with the fast translational diffusion of Na^+^ ions^35^. Although we observed no obvious translational diffusion of Li^+^ ions in our FPMD calculations because of the limited simulation time (12 ps) and the unit cell size (136 atoms), the significant rotational motions of [MoH_9_]^3−^ ions, as observed in Fig. 4a, suggest the potential fast lithium ion conduction even at room temperature. It should be noted that given the quantum mechanical tunnelling of the H atoms, the rotational motions of hydride complexes may be sustained at extremely low temperature at which thermal diffusion is supressed.

Although the hydride complexes with ninefold H-coordination were discovered in 1964^18^, they are very rare and their properties have not been fully clarified. Therefore, our findings will facilitate the discovery of latent functionalities, such as those described in this study and will lead to renewed interest in complex transition metal hydrides.

## Methods

### DFT calculations

The ground-state crystal structures of the Li–Mo–H, Li–W–H, Li–Nb–H and Li–Ta–H systems were explored using the structures of existing compounds as references. This exploration was performed using first-principles DFT calculations as implemented in the Vienna ab initio simulation package (VASP)^36^,^37^. We used a plane-wave basis and the projector augmented wave method^38^,^39^ within the generalized gradient approximation with the Perdew–Burke–Ernzerhof exchange-correlation functional^40^. For Li_5_MoH_11_, we assumed that the structure has a metal framework similar to that of CaNi_5_ because of the similarity in their metal stoichiometries (Ca and Ni were replaced by Mo and Li, respectively, in the doubled CaNi_5_ structure along the *c*-axis). We used nine H atoms (18 H atoms per formula unit) to coordinate the Mo atoms in the fashion of a tricapped trigonal prism, and placed two H atoms (four H atoms per formula unit) in the largest tetragonal site comprising four Li atoms. For Li_6_NbH_11_, an additional Li atom (two Li atoms per formula unit) was placed at the centre of the largest trigonal antiprism comprising six H atoms in Li_5_MoH_11_ to accommodate the 6:1 stoichiometry between the Li and transition metals. We assumed that Li_5_WH_11_ and Li_6_TaH_11_ have isostructures to Li_5_MoH_11_ and Li_6_NbH_11_, respectively. Next, full structure relaxations, including those for the lattice parameters, shapes and internal coordinates, were performed. Subsequently, phonon calculations were performed to verify that the relaxed structures were at the true minimum. When imaginary phonon frequencies were observed, we slightly displaced the atoms along the directions of the eigenvectors of the imaginary modes and further relaxed the structures to eliminate them. This procedure was performed until the ground state was reached. This was done using well-converged plane-wave basis sets with a cut-off energy of 800 eV. An 8 × 8 × 4 grid was used for the ***k***-point sampling of the Brillouin zone.

### FPMD calculations

The room-temperature phase of Li_5_MoH_11_ was examined using FPMD calculations in the *NPT* ensemble at 298 K with a 2 × 2 × 1 supercell (136 atoms) of the primitive cell. We employed a well-converged cut-off energy of 600 eV with ***k***-point sampling at the Γ-point. The simulation temperature was set to 298 K, which was controlled by a Langevin thermostat^41^ with a friction coefficient of 10 ps^−1^ for all atoms. The simulation pressure was controlled by a Parrinello–Rahman barostat^42^,^43^, where a friction coefficient of 10 ps^−1^ were used for the lattice degrees of freedom. The total simulation time was 12 ps with a timestep of 0.5 fs.

### Theoretical ND profile

The integrated time-of-flight (TOF) ND peak intensity *I(**K***_*n*_) at the FPMD step *n* was calculated from the FPMD trajectory using the following equation:


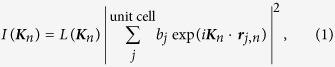


where ***K***_*n*_ is the reciprocal lattice vector, *L(**K***_*n*_) is the Lorentz factor, *b*_*j*_ is the neutron-scattering length of atom *j*, and ***r***_*j,n*_ is the position of atom *j*. The ND profile was obtained by integrating the discrete ND peaks broadened by a Gaussian function with a width of 0.02 Å over a range from 0.8 fs to 12 ps, where the unit cell volume is well converged (see Fig. 4b).

### Syntheses

High-purity metal powders of Mo (99.99%, Sigma-Aldrich), W (99.99%, Sigma-Aldrich), Nb (99.9%, Mitsuwa Chemicals Co., Ltd.) and Ta (99.9%, Mitsuwa Chemicals Co., Ltd.) were mixed with LiH (95%, Sigma-Aldrich) at molar ratios of 1:6 (Li–Mo–H and Li–W–H) and 1:8 (Li–Nb–H and Li–Ta–H) by mechanical milling at 400 rpm for 4 h under a 0.1-MPa Ar atmosphere. The mixtures were compacted into pellets (diameter and thickness = 1.0 mm) and encapsulated in sample capsules comprising pyrolytic boron nitride. The capsules were loaded together with a hydrogen source (AlH_3_) into NaCl capsules, which were used to seal the hydrogen fluid that evolved from AlH_3_. The NaCl capsules were pressurized to 5 GPa at room temperature using a multi-anvil high-pressure apparatus, followed by subsequent heating at 923 K (Li–Nb–H), 973 K (Li–Mo–H) and 1023 K (Li–W–H and Li–Ta–H) for 24 h (Li–W–H and Li–Ta–H) and 48 h (Li–Mo–H and Li–Nb–H). A similar procedure was used to synthesize the deuteride analogue Li_5_MoD_11_, where LiD and AlD_3_ were used in place of LiH and AlH_3_.

### ND

Neutron scattering experiments of Li_5_MoD_11_ (41 mg) were performed for an exposure time of 8 h at room temperature under He on the NOVA high-intensity total diffractometer installed at the 500 kW spallation neutron source at the Materials and Life Science Experimental Facility (MLF) Japan Proton Accelerator Research Complex (J-PARC). The sample was filled in a cylindrical sample container with an external diameter of 3.0 mm and thickness of 0.1 mm. Rietveld analysis was performed using the General Structure Analysis System (GSAS) software and the graphical interface EXPGUI (version 1.80)^44^ on the ND data, with *d* = 0.25 Å–6.16 Å obtained at room temperature from detectors of medium resolution (Δ*d*/*d* ~ 0.6%) at the scattering angle 2*θ* = 72°–108°. The profile function developed by Von Dreele *et al*.^45^ was used for performing Rietveld analysis. The background was modeled using the 10-terms Chebyschev polynomial function model in GSAS. The distances of Mo–D in the complex anion [MoD_9_]^3−^ and Li–D in the tetrahedral site in Li_5_MoD_11_ were soft constrained to 1.75 Å. The constraints were weighted such that they imposed only a minor deterioration of the fit to the experimental data. ND peaks of an unknown phase were indexed by a monoclinic unit cell with *a* ≈ 9.79 Å, *b* ≈ 7.03 Å, *c* ≈ 5.55 Å and *β* ≈ 105.7° using the indexing programs TREOR97^46^ and PIRUM^47^. Based on *hkl* extinction rules, the space group for the unknown phase was predicted to be *P*2_1_/*m* (No. 11). Because it was not possible to determine the atomic positions in the unknown phase, Le Bail fitting was simultaneously performed for the unknown phase in Rietveld analysis.

## Additional Information

**How to cite this article**: Takagi, S. *et al*. Formation of novel transition metal hydride complexes with ninefold hydrogen coordination. *Sci. Rep.* **7**, 44253; doi: 10.1038/srep44253 (2017).

**Publisher's note:** Springer Nature remains neutral with regard to jurisdictional claims in published maps and institutional affiliations.

## Supplementary Material

Supplementary Information

## Figures and Tables

**Table 1:** Enthalpy changes Δ*H*_r_ and entropy changes Δ*S*_r_ of the conceivable hydrogenation reactions.

Hydrogenation reaction	Δ*H*_r_ (kJ/mol)	Δ*S*_r_ (J/mol · K)
5LiH + Mo + 3H_2_ → Li_5_MoH_11_	−105	−254
5LiH + W + 3H_2_ → Li_5_WH_11_	−128	−264
6LiH + NbH_0.65_ + 2.175H_2_ → Li_5_MoH_11_	−91	−120
6LiH + TaH_0.65_ + 2.175H_2_ → Li_5_TaH_11_	−117	−151

**Table 2:** Experimental lattice parameters determined by X-ray diffraction (XRD) measurements at room temperature.

Sample	Space group	*a* (Å)	*c* (Å)
Li–Mo–H	*P*6_3_*cm* (185)	5.591(1) (5.351)	11.295(6) (11.035)
Li–W–H	*P*6_3_*cm* (185)	5.544(2) (5.357)	11.415(6) (11.038)
Li–Nb–H	*P*6_3_*cm* (185)	5.638(2) (5.482)	11.612(6) (11.420)
Li–Ta–H	*P*6_3_*cm* (185)	5.510(1) (5.484)	11.813(6) (11.401)

The corresponding density functional theory (DFT) lattice parameters are given in parentheses.

**Table 3:** Structural parameters of Li_5_MoH_11_ determined by neutron diffraction (ND) measurements at room temperature.

Element	Wyckoff notation	*x*	*y*	*z*	occupancy	100 × *U*_iso_ (Å)
Mo1	2*a*	0	0	−0.018(3)	1.0	4.0
Li1	4*b*	1/3	2/3	0.0780	1.0	1.0
Li2	6*c*	1/2	0	0.2943	1.0	1.0
D1	12*d*	0.3011(11)	0.0000(11)	−0.018(3)	0.3	3.77(27)
D2	12*d*	0.1819(11)	0.3546(9)	0.0234(30)	0.3	3.77(27)
D3	12*d*	0.1915(15)	0.0004(18)	0.1068(30)	0.3	3.77(27)
D4	12*d*	0.1819(11)	0.3546(9)	0.441(4)	0.3	3.77(27)
D5	12*d*	0.1915(15)	0.0004(18)	0.3569(30)	0.3	3.77(27)
D6	4*b*	1/3	2/3	0.2324(7)	0.3	2.62(28)

The deuteride occurs in a hexagonal structure of space group *P*6*cc* (No. 184) with lattice parameters *a* = 5.5053(8) Å and *c* = 11.2095(19) Å. The isotropic temperature factor *U*_iso_ for Li and Mo are not allowed to refine.
