# Supplementary material for: Formation of novel transition metal hydride complexes with ninefold hydrogen coordination
Source: Sci Rep. 2017 Mar 13;7:44253. doi: 10.1038/srep44253 (PMC5347150; doi:10.1038/srep44253)
Supplement: Supplementary Information [file srep44253-s1.doc]

**Formation of novel transition metal hydride complexes with ninefold hydrogen coordination**

Shigeyuki Takagi^1^, Yuki Iijima^1^, Toyoto Sato^1^, Hiroyuki Saitoh^2^, Kazutaka Ikeda^3^, Toshiya Otomo^3^, Kazutoshi Miwa^4^, Tamio Ikeshoji^1^ and Shin-ichi Orimo^1,5,*^

^1^Advanced Institute for Materials Research, Tohoku University, Sendai 980-8577, Japan

^2^Quantum Beam Science Research Directorate, National Institute for Quantum and Radiological Science and Technology, Hyogo 679-5148, Japan

^3^Institute for Materials Structure Science, High Energy Accelerator Research Organization, Tsukuba 305-0801, Japan

^4^Toyota Central R&D Laboratories, Inc., Nagakute 480-192, Japan

^5^WPI-Advanced Institute for Materials Research, Tohoku University, Sendai 980-8577, Japan

**High-pressure phases of Li_5_MoH_11_.** Starting from the hexagonal *P*6_3_*cm* structure of Li_5_MoH_11_ obtained by DFT calculations at ambient pressure, we systematically explored the high-pressure phases, and found 15 different structures besides the one stable at ambient pressure without any lattice instabilities under compression (see Methods for the procedure, and Figs. S2–S16 and Tables S1–S15 for the crystal structures).

Figure S1a shows the thermodynamic stability of the three energetically competing phases of Li_5_MoH_11_, as a function of compression rate, 100(*V*_0_–*V*)/*V*_0_, where *V*_0_ denotes the volume at ambient pressure (see Fig. S17 for the thermodynamic stability of all the structures). The *P*6_3_*cm* structure undergoes the first phase transition to the monoclinic *Cc* structure at 5 GPa. The *Cc* structure retains the ninefold H-coordination and two isolated H^–^ ions, as in the inset at the bottom left of Fig. S1a, and the electronic structure is still insulating (see Fig. S18 for the electronic structure). Then, the second phase transition from the *Cc* to *Pc* structures occurs at 94 GPa. In the latter phase, while a quarter of two isolated H^–^ ions (H_0.5_ per formula unit) remains in the Li lattice, a half of the H^–^ ions forms the segregated H_2_ units ((H_2_)_0.5_ per formula unit). More interestingly, the remaining quarter of H^−^ ions (H_0.5_ per formula unit) participate in the formation of more hydrogen-rich three corner-sharing MoH_11_ units (MoH_9+0.5_ per formula unit) with elevenfold H-coordination, as in the inset at the bottom right of Fig. S1a. The resulting H sublattice (Fig. S1b) induces an insulator-to-metal transition with substantial contribution from H 1*s* states at *E*_F_ (Fig. S1c). The participation of H^−^ ions in the formation of hydride complexes would result from the large ionic radius and hardness of the closed-shell H^−^ ion, which significantly destabilize the insulating phase under compression, leading to hybridization with other elements to reduce the size. The mechanism would be quite general in principle, and should be applicable to all of the compounds synthesized in this study.

**Methods.** The crystal structure, electronic structure and energetics of Li_5_MoH_11_ under compression were systematically investigated using first-principles calculations in the following way: One first performed structure optimizations of Li_5_MoH_11_ with compressed volumes ranging from 80 to 240 Å^3^ per formula unit with 10 Å^3^ increments in between, where we relaxed the internal coordinates of all the atoms and lattice shape at each volume. The subsequent phonon calculations exhibit several imaginary phonon frequencies in the structures with a volume range of 80–140 Å^3^. Then, we eliminated the imaginary phonon frequencies by displacing slightly the atoms along the directions of eigenvectors of the imaginary modes and further relaxed the structures. This procedure was carried out toward all of the imaginary phonon frequencies until the ground states were reached. In this way, we successfully obtained 15 different structures besides the one stable at ambient pressure, as depicted in Figs. S4–S18 (the structural parameters are summarized in Tables S6–S20). We then evaluated the total energies of the structures as a function of the cell volume, where the internal coordinates of all the atoms and lattice shapes were relaxed at each volume. The calculated energies were fitted to the third-order Birch-Murnaghan equation of states (EoS)^[S1,S2]^ in order to obtain the bulk moduli, which were used to estimate the applied pressures at the volumes. All calculations were done using well-converged plane-wave basis sets with a cut-off energy of 800 eV. An 8 × 8 × 4 grid was used for the *k*-point sampling of the Brillouin zone.

**References**

1. F. D. Murnaghan, *Proc. Natl. Acad. Sci. U. S. A.* **1944**, *30*, 244–247.
2. F. Birch, *Phys. Rev.* **1947**, *71*, 809–824.

**Figures**

**Figure S1.** (a) Thermodynamic stability of the three energetically competing phases of Li_5_MoH_11_ as a function of compression rate. The enthalpy variation of the hexagonal *P*6_3_*cm* structure is taken as a reference. The vertical dashed lines indicate the compression rates corresponding to the first-order phase transitions. The insets are the schematic depiction of the frameworks of the hydride complexes and the isolated H atoms in *P*6_3_*cm*/*Cc* (left) and *Pc* (right) phases. (b) Total electronic DOS (top) and H 1*s* projection (bottom) for the metallic *Pc* phase of Li_5_MoH_11_ at 94 GPa. The energy zero is set at the valence-band maximum. (c) Crystal structure of the high-pressure phase of Li_5_MoH_11_ with space group *Pc* (No. 7). The black solid lines indicate the unit cell boundary.


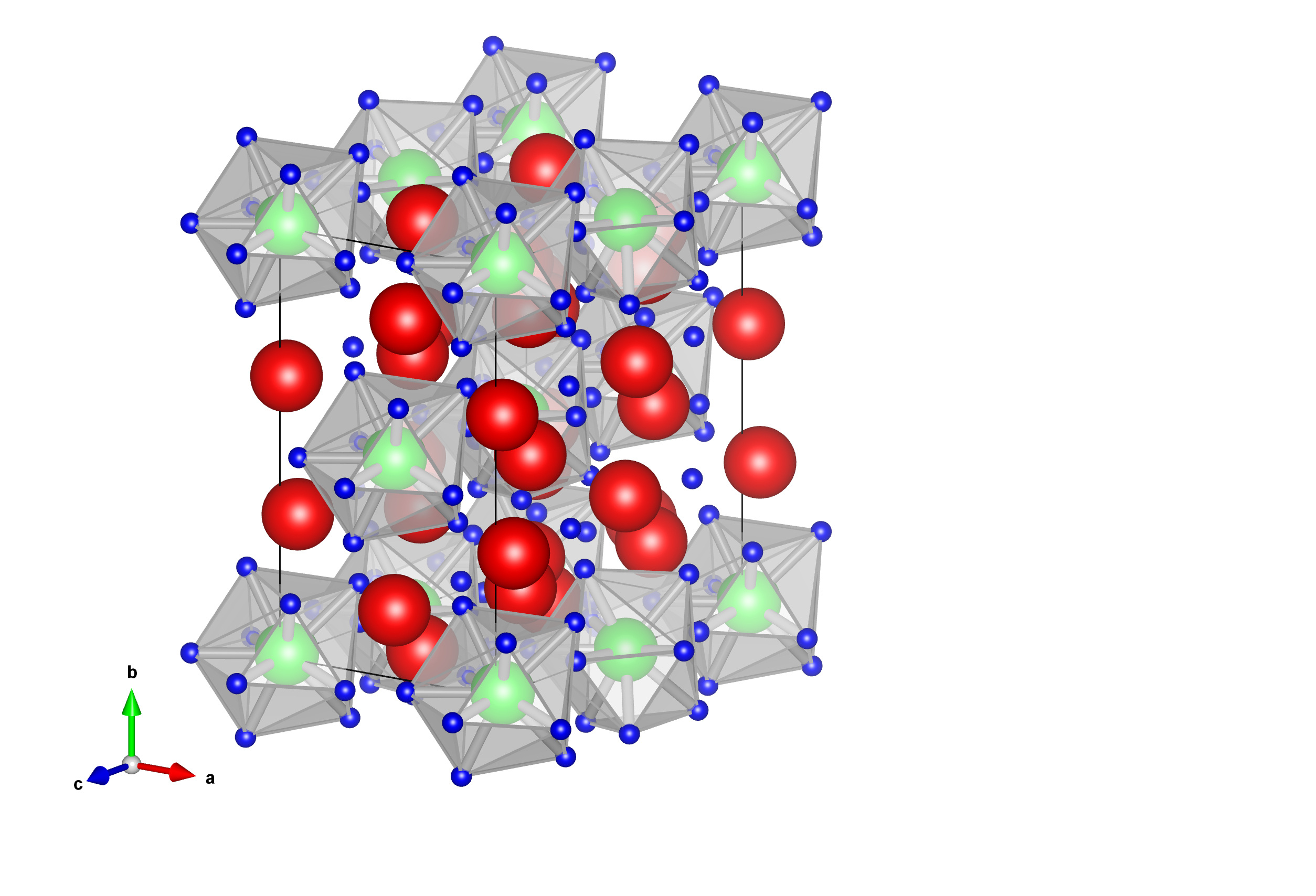


**Figure S2.** Crystal structure of high-pressure phase of Li_5_MoH_11_ with space group *Cc* (9) (the *Cc* structure in the text).


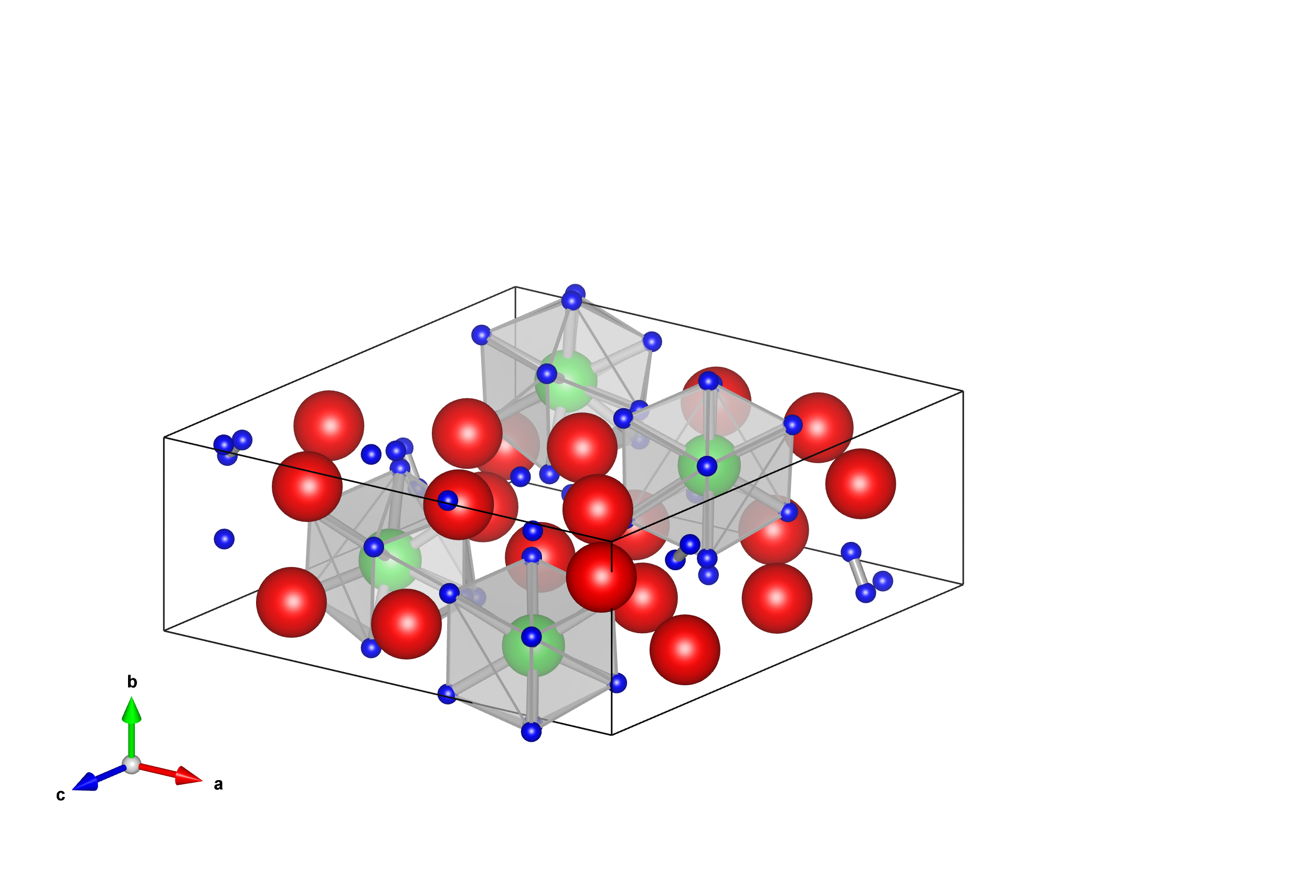


**Figure S3.** Crystal structure of high-pressure phase of Li_5_MoH_11_ with space group *Pc* (7) (the *Pc* structure in the text).


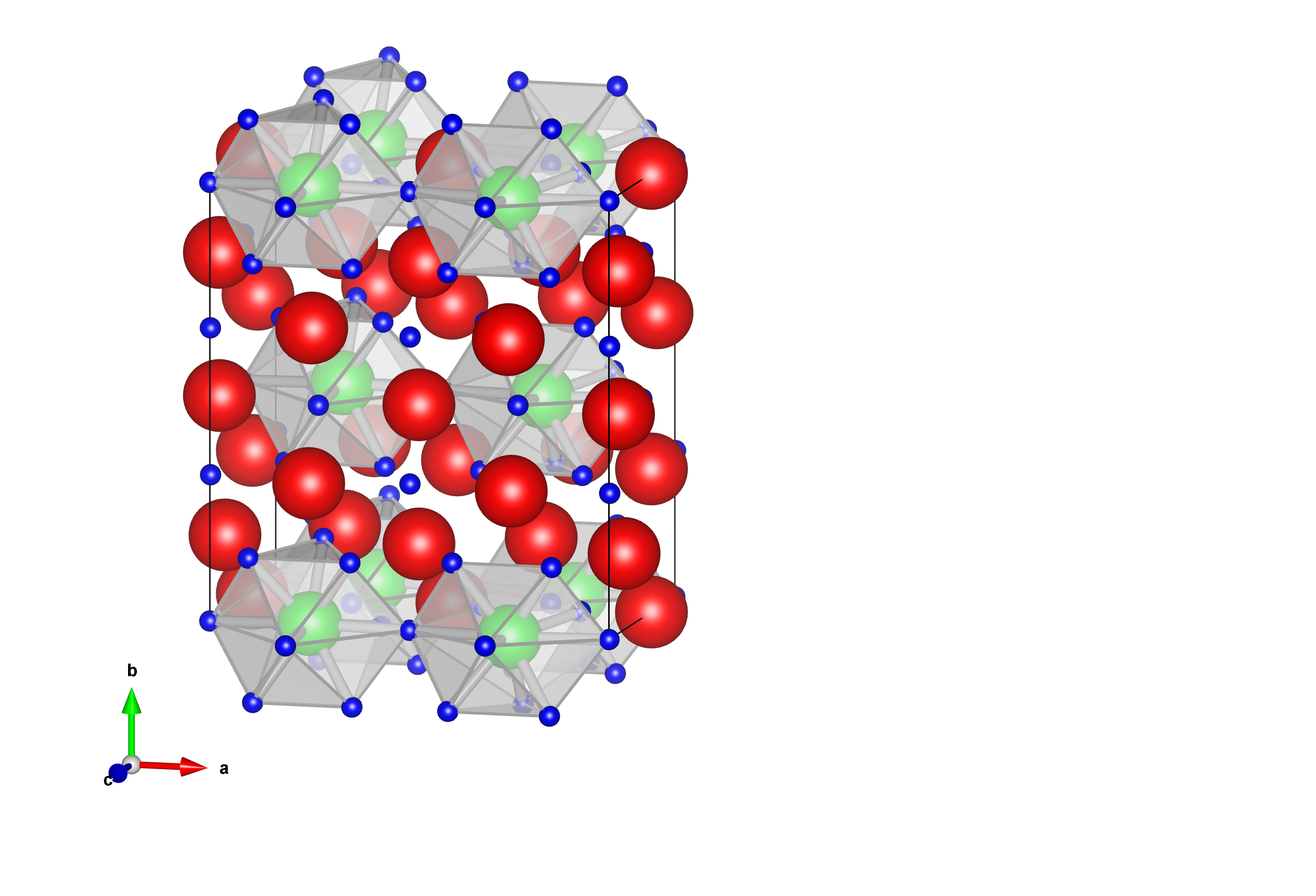


**Figure S4.** Crystal structure of high-pressure phase [1] of Li_5_MoH_11_ with space group *Ama*2 (40).


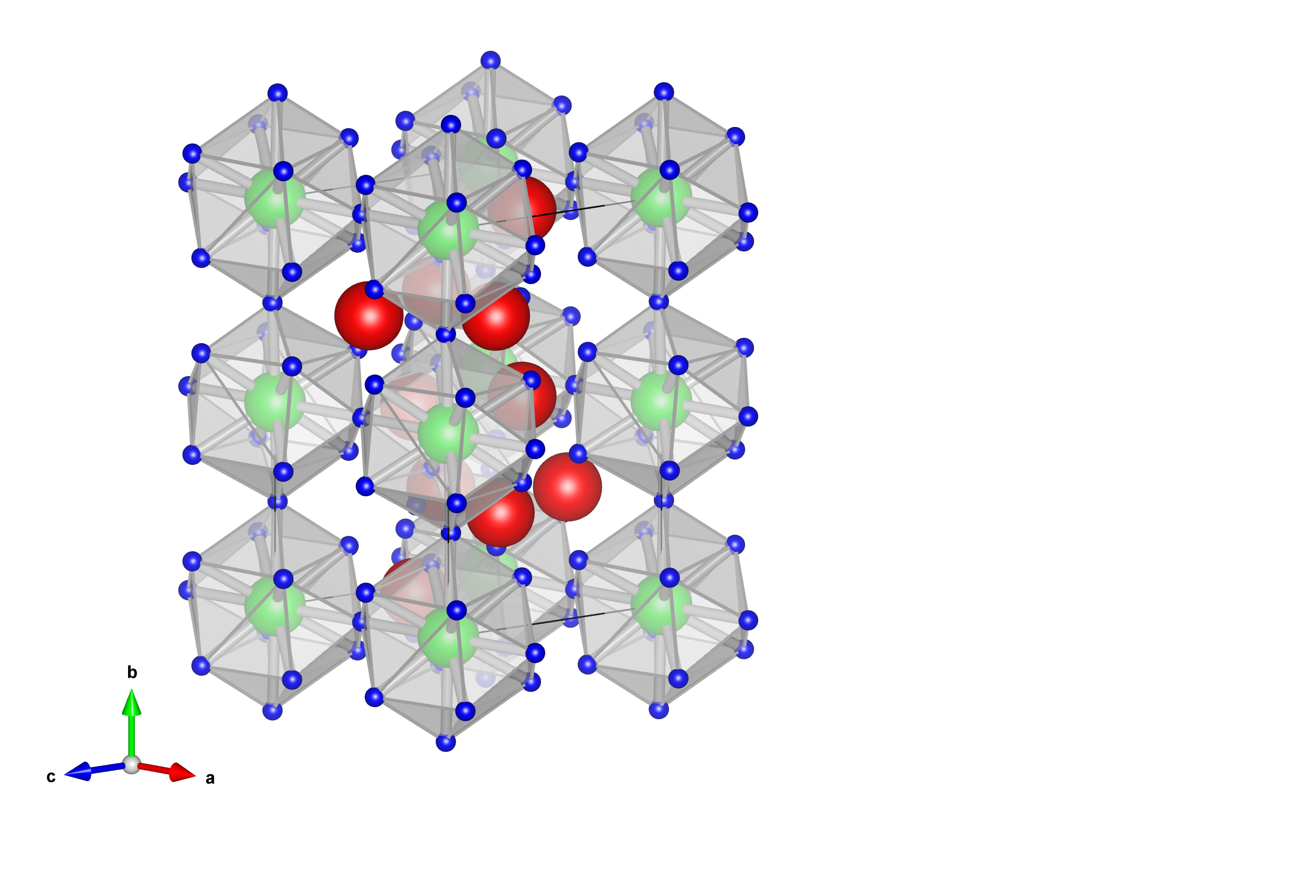


**Figure S5.** Crystal structure of high-pressure phase [2] of Li_5_MoH_11_ with space group *P*2_1_/*m* (11).


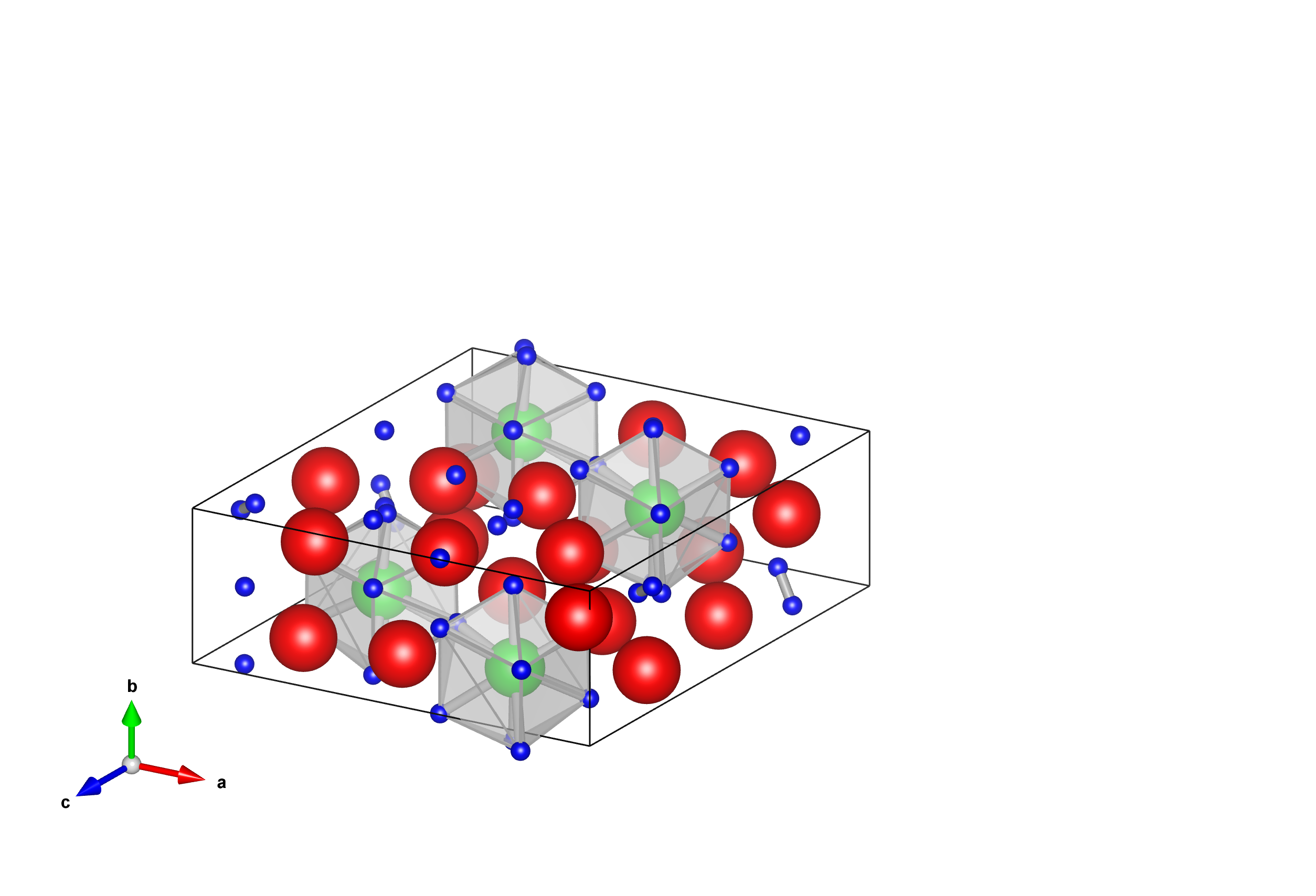


**Figure S6.** Crystal structure of high-pressure phase [3] of Li_5_MoH_11_ with space group *Pc* (7).


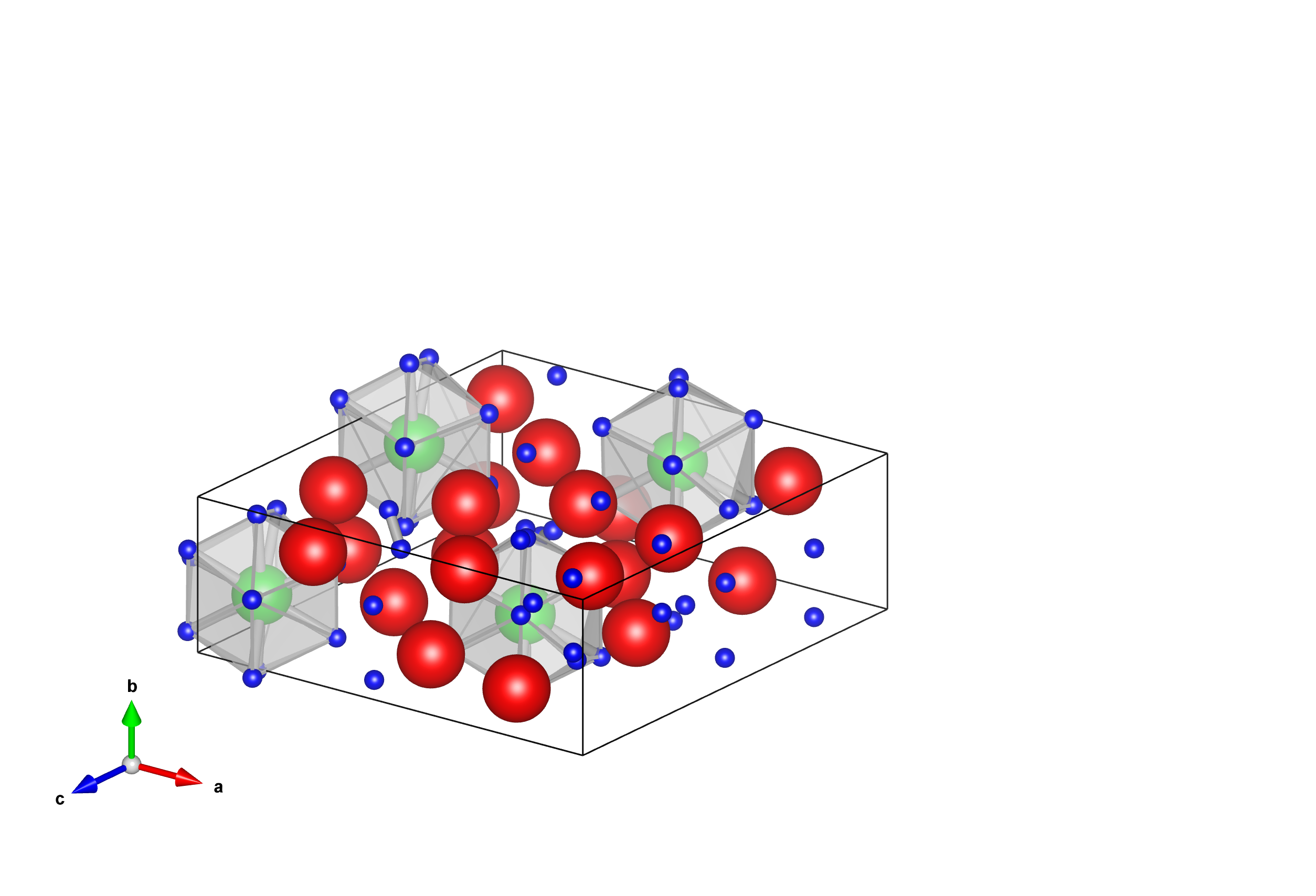


**Figure S7.** Crystal structure of high-pressure phase [4] of Li_5_MoH_11_ with space group *Pc* (7).


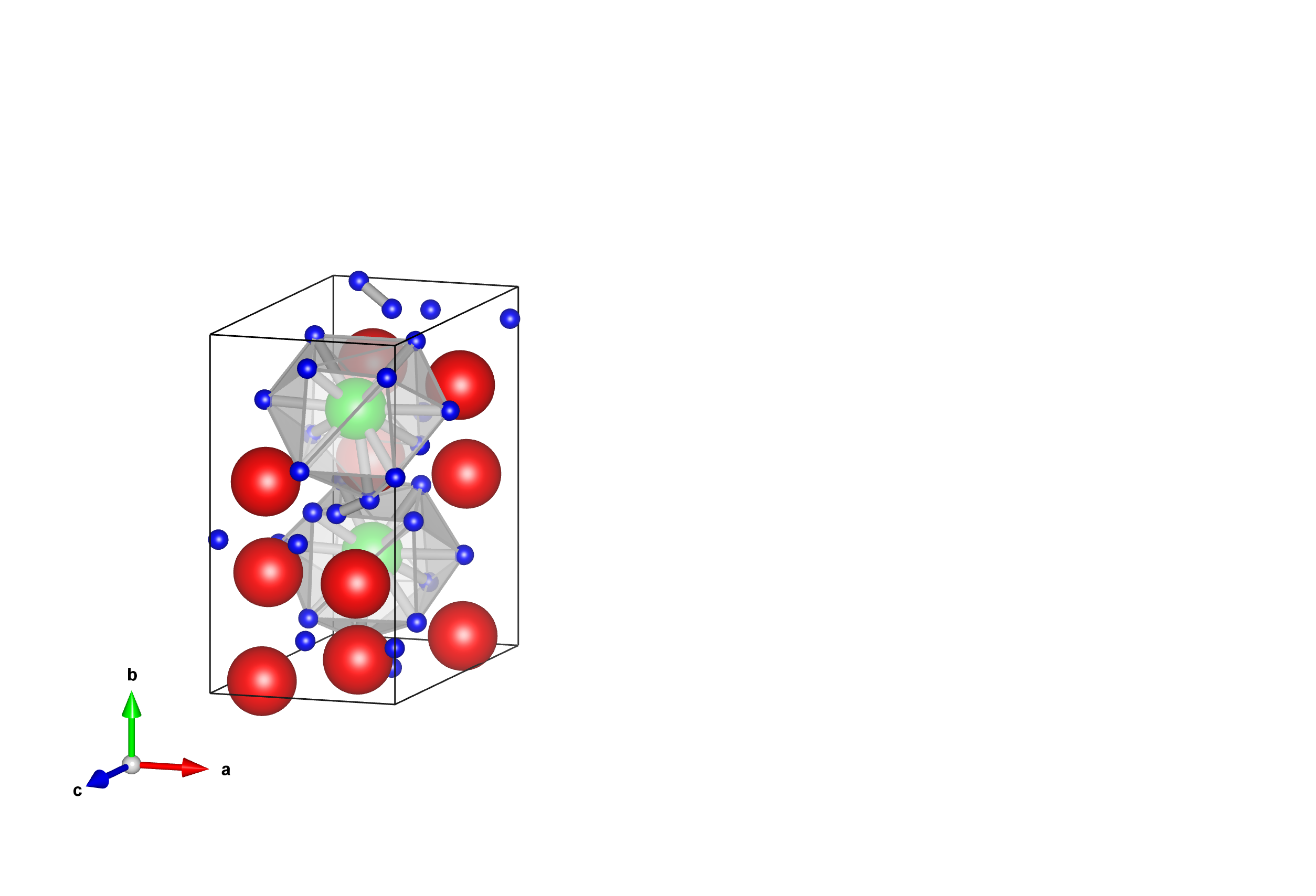


**Figure S8.** Crystal structure of high-pressure phase [5] of Li_5_MoH_11_ with space group *P*2_1_ (4).


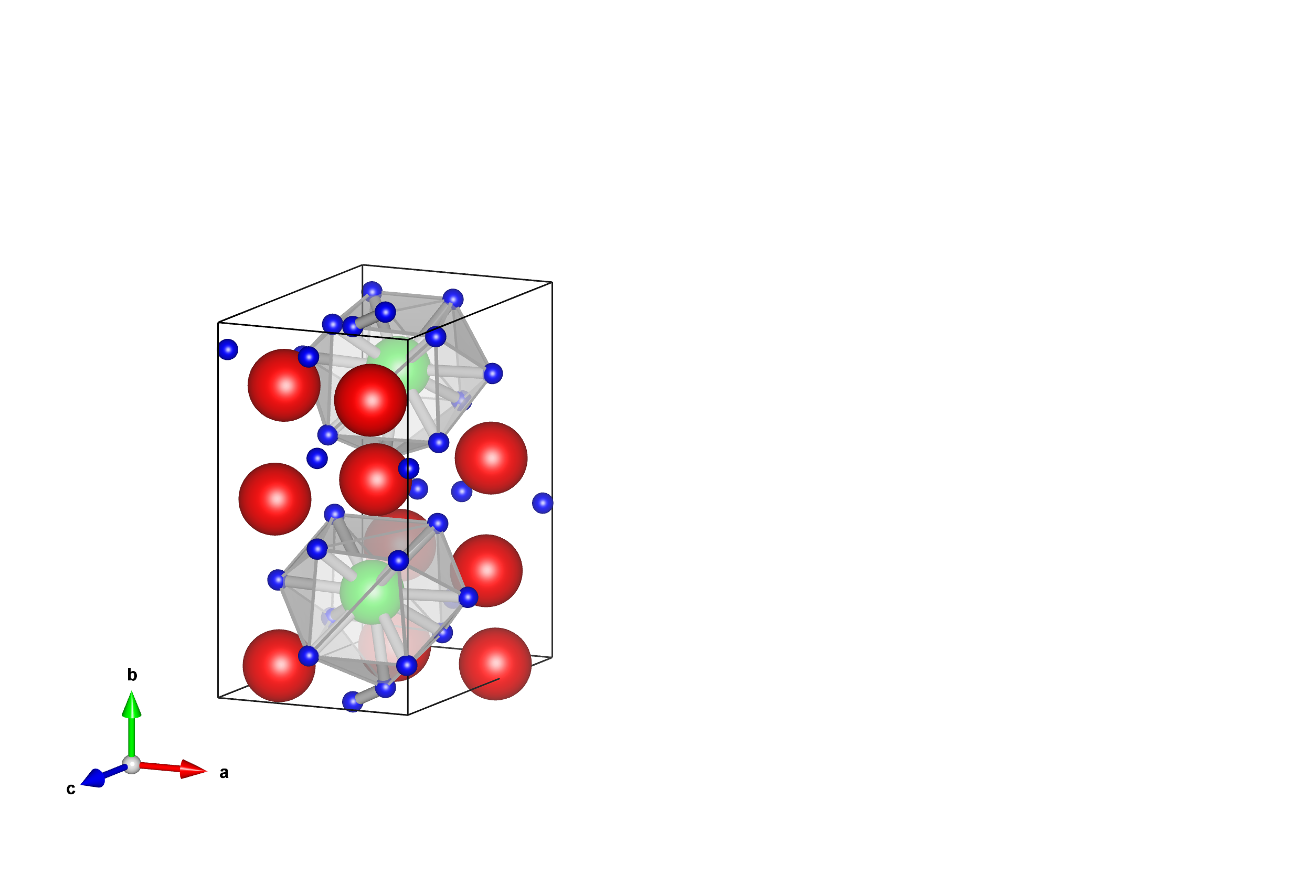


**Figure S9.** Crystal structure of high-pressure phase [6] of Li_5_MoH_11_ with space group *P*2_1_ (4).


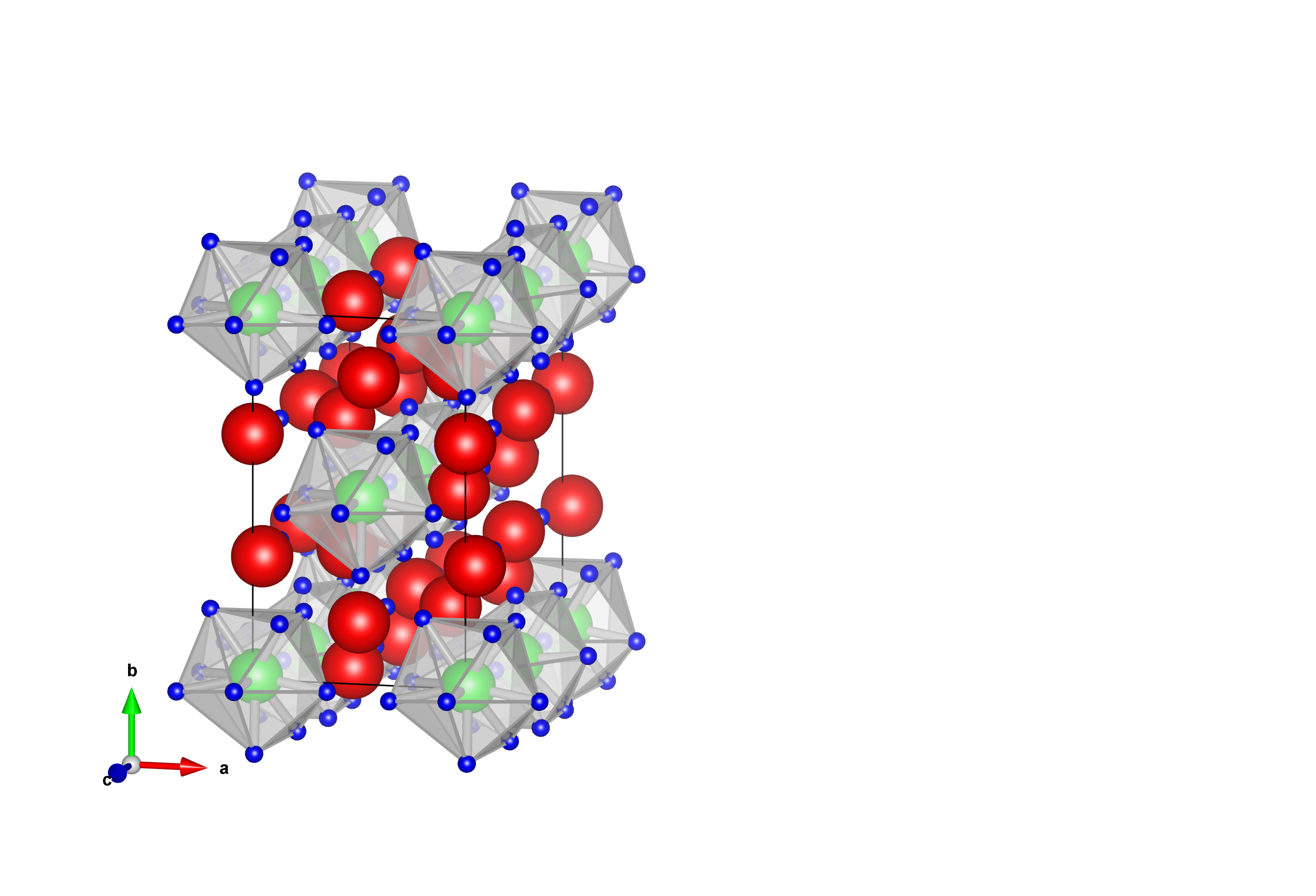


**Figure S10.** Crystal structure of high-pressure phase [7] of Li_5_MoH_11_ with space group *Cc* (9).


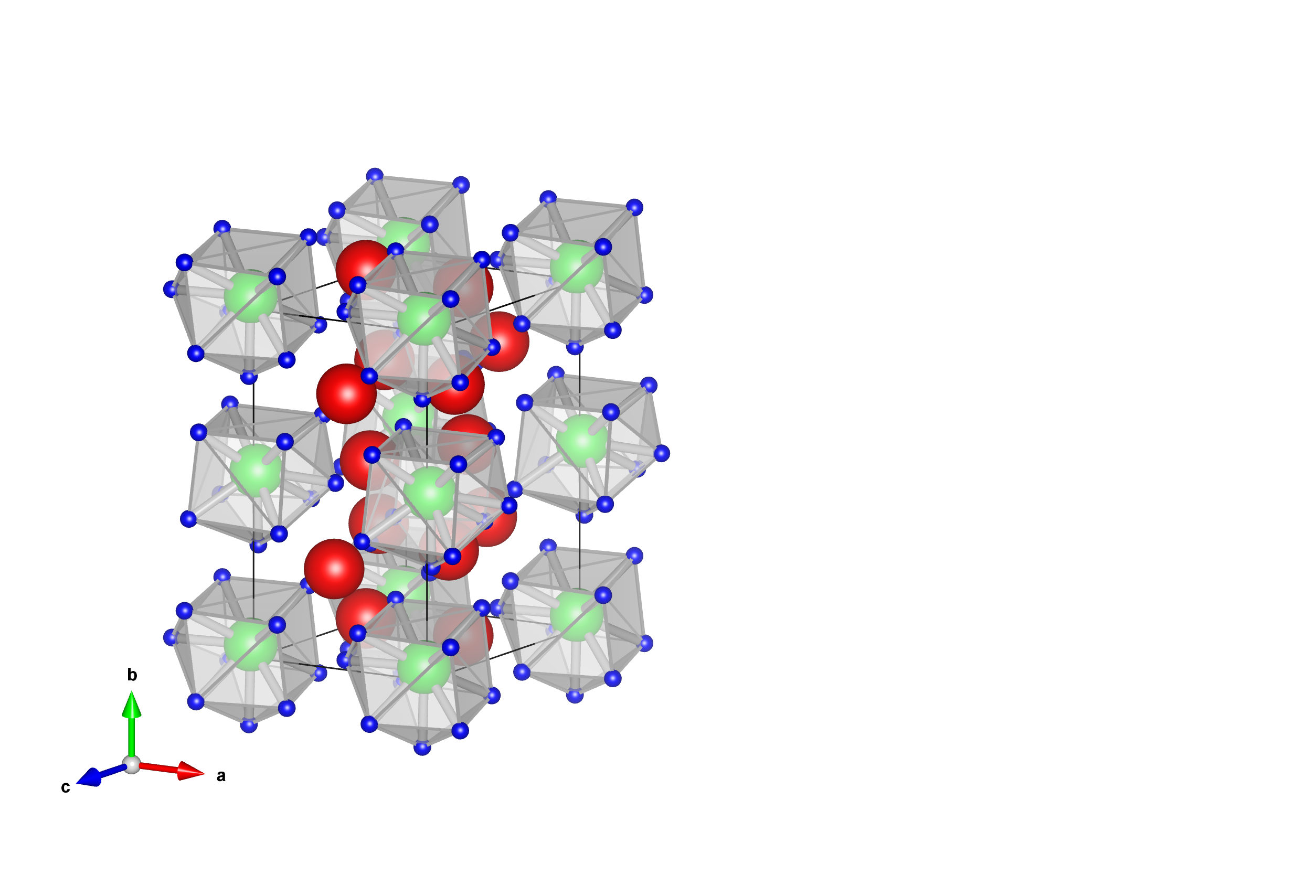


**Figure S11.** Crystal structure of high-pressure phase [8] of Li_5_MoH_11_ with space group *P*2_1_ (4).


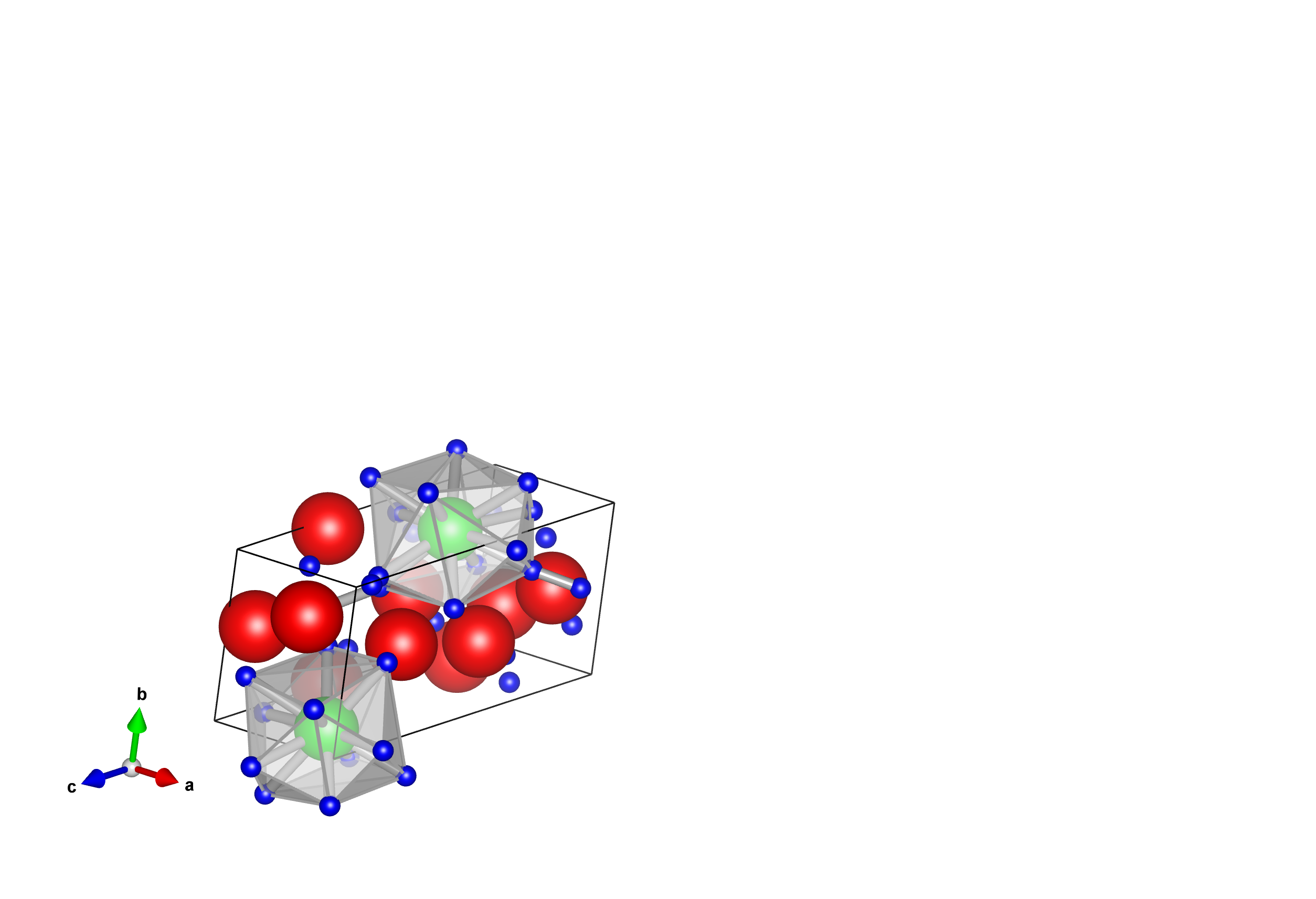


**Figure S12.** Crystal structure of high-pressure phase [9] of Li_5_MoH_11_ with space group *P*1 (1).


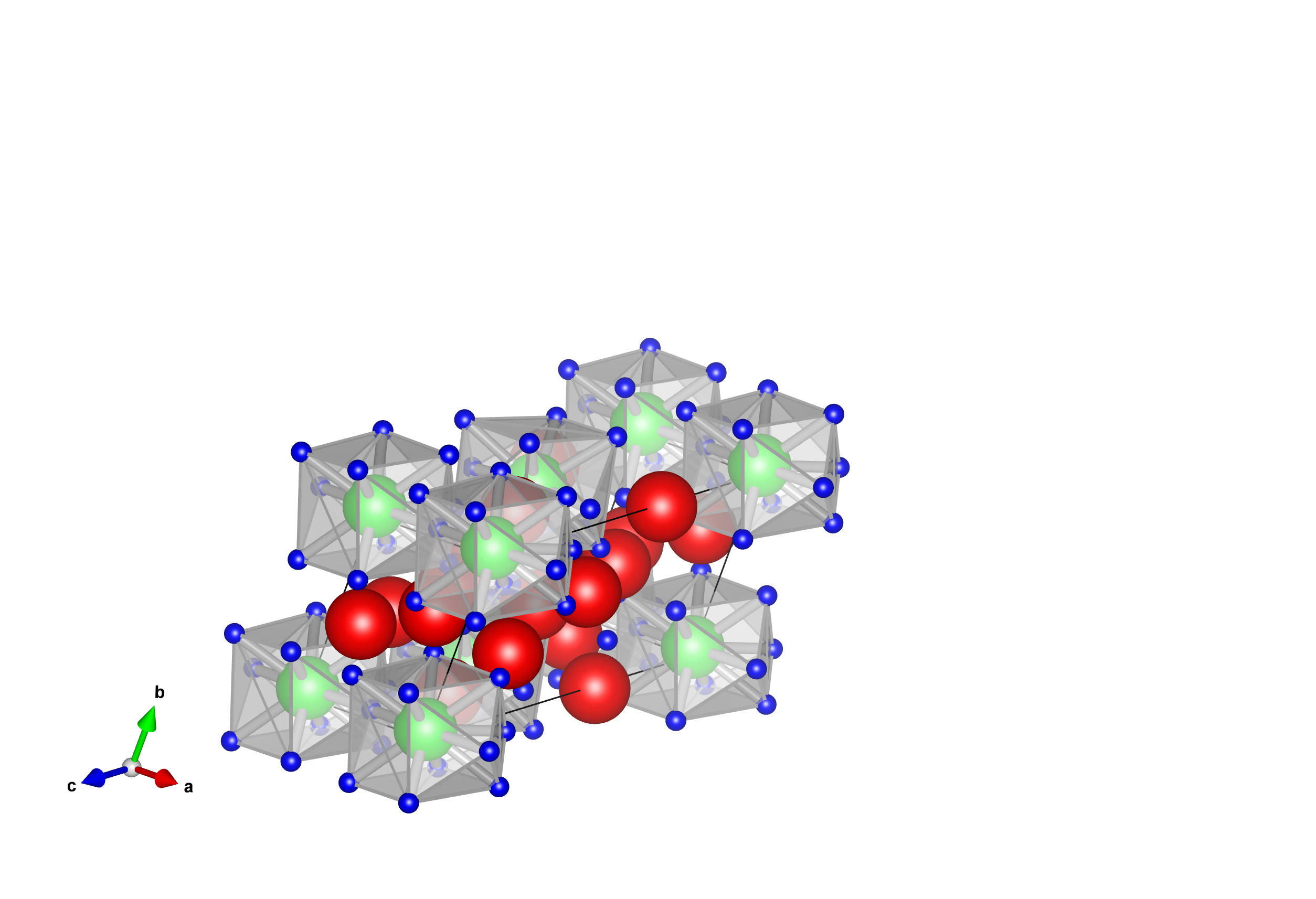


**Figure S13.** Crystal structure of high-pressure phase [10] of Li_5_MoH_11_ with space group *P*1 (1).


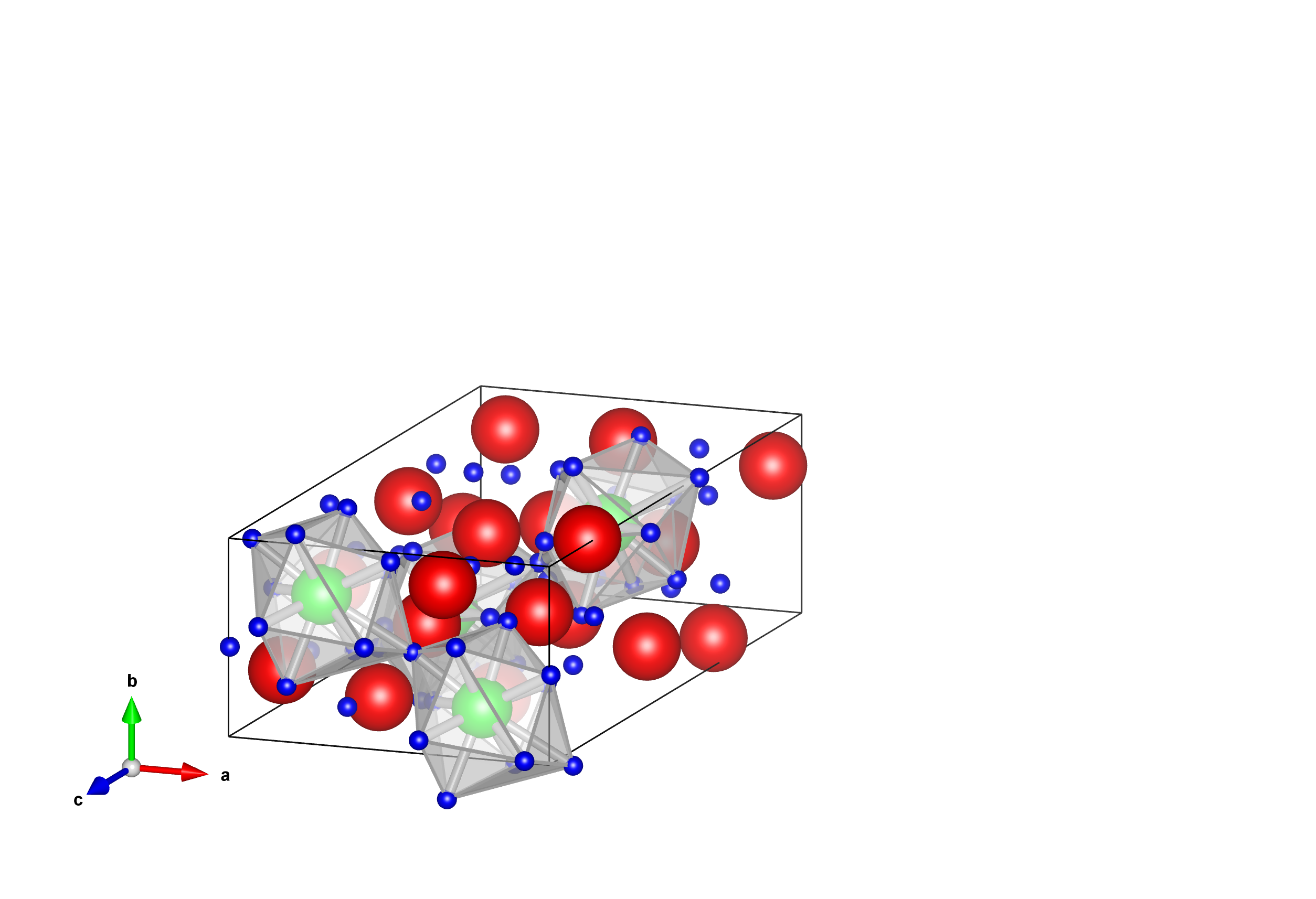


**Figure S14.** Crystal structure of high-pressure phase [11] of Li_5_MoH_11_ with space group *Cc* (9).


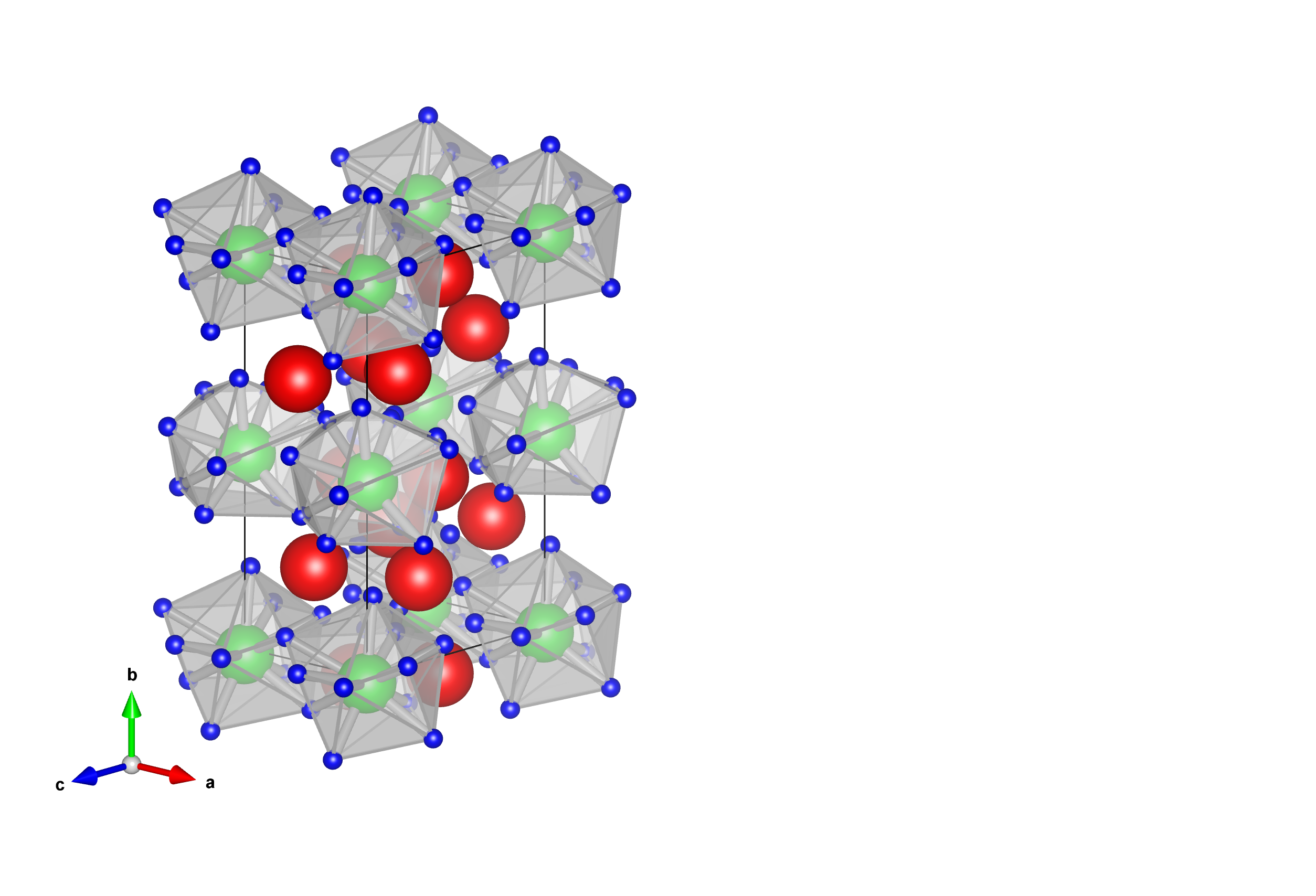


**Figure S15.** Crystal structure of high-pressure phase [12] of Li_5_MoH_11_ with space group *P*2_1_ (4).


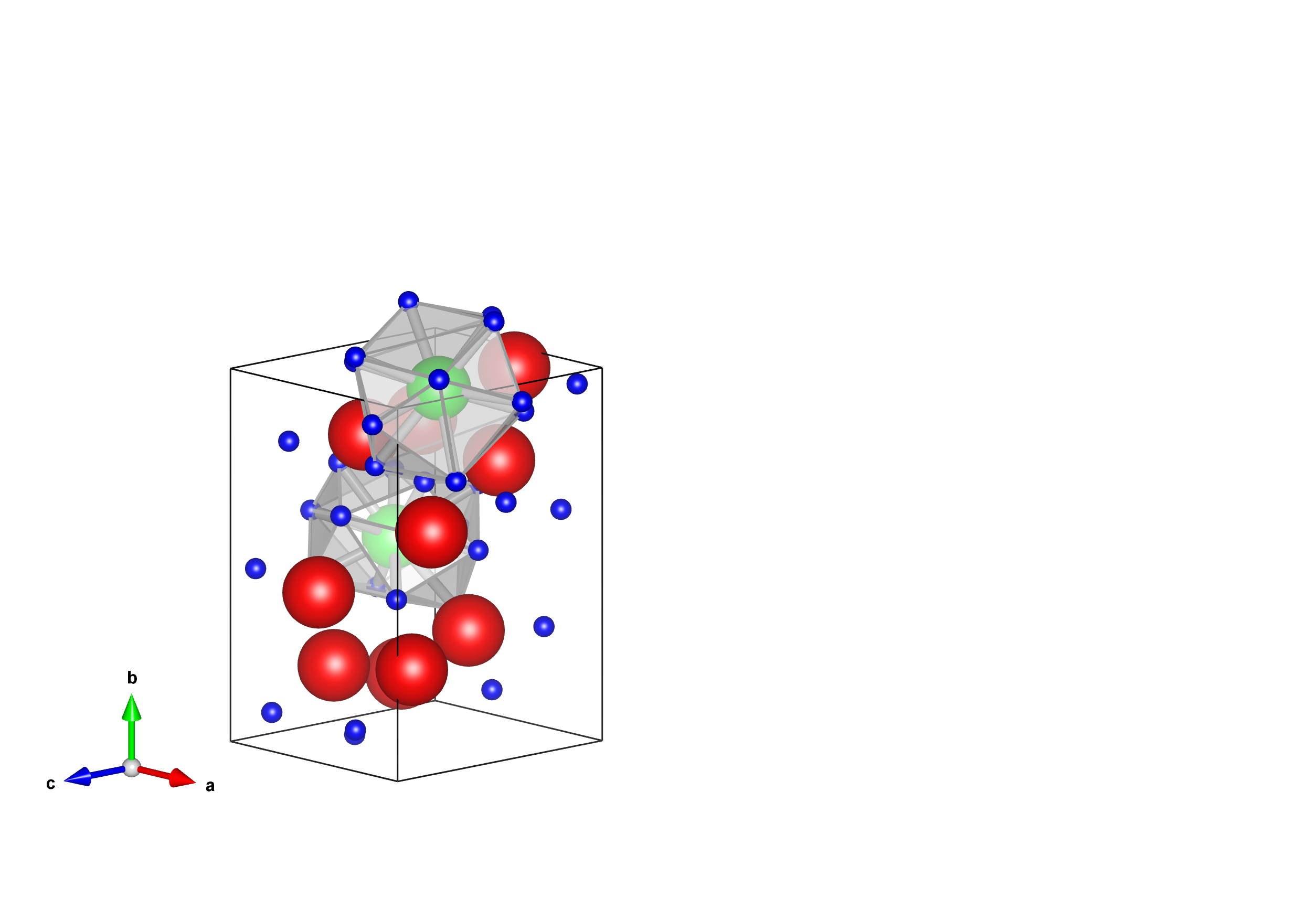


**Figure S16.** Crystal structure of high-pressure phase [13] of Li_5_MoH_11_ with space group *P*2_1_ (4).

**Figure S17.** Thermodynamic stability of the 15 different phases as a function of compression rate. The enthalpy variation of the hexagonal *P*6_3_*cm* structure the most stable at ambient pressure is taken as a reference.


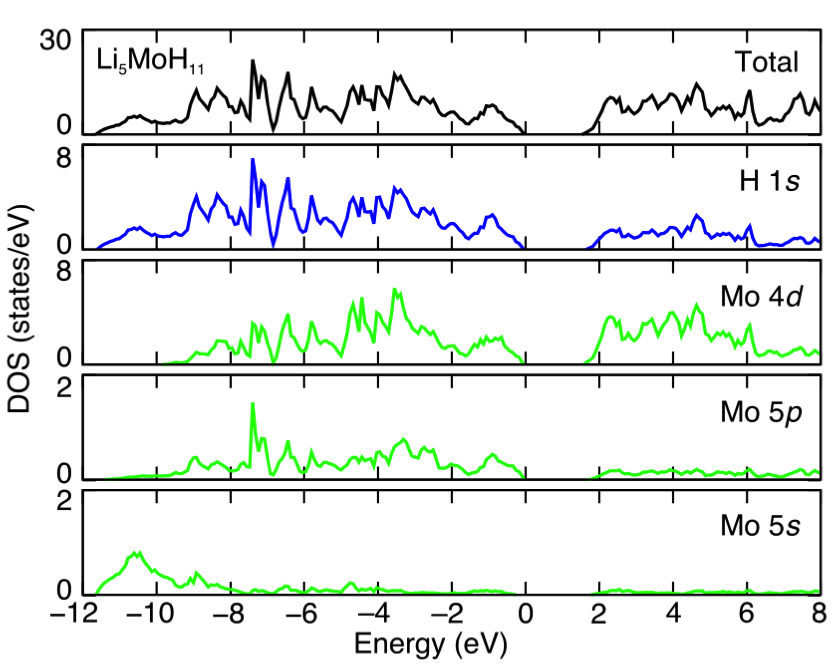


**Figure S18.** Total electronic DOS (top), H 1*s* (second panel from the top), Mo 4*d* (middle), Mo 5*p* (second panel from the bottom) and Mo 5*s* (bottom) projections for the insulating *Cc* phase of Li_5_MoH_11_ at 85 GPa. The energy zero is set at the valence-band maximum.


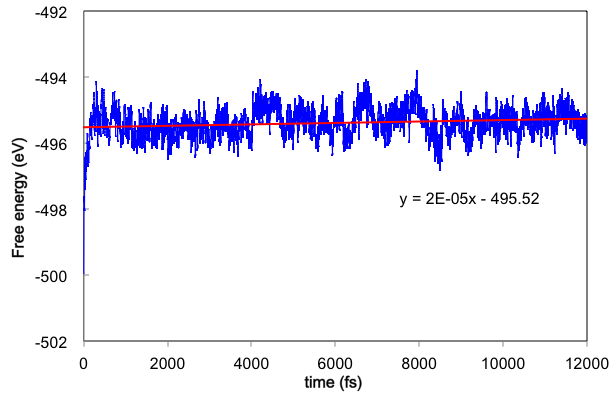


**Figure S19.** Evolution of the free energy of Li_5_MoH_11_ in FPMD calculations in the *NpT* emsenble at 298 K.

**Tables**

**Table S1: Structural parameters of high-pressure phase of Li_5_MoH_11_ the most stable over a range of compression rate of 12.1–53.5 % (corresponding to the *Cc* structure in the main text). The lattice cell is an orthorhombic with space group *Cc* (No. 9) and lattice parameters are *a* = 4.549 Å, *b* = 7.685 Å, *c* = 7.413 Å and *β* = 90.023˚.**

| Atom | Wyckoff notation | *x* | *y* | *z* |
| --- | --- | --- | --- | --- |
| Mo1 | 4*a* | 0.0013 | 0.0034 | –0.0261 |
| Li1 | 4*a* | 0.0446 | 0.3300 | –0.0336 |
| Li2 | 4*a* | 0.4923 | 0.1473 | –0.0332 |
| Li3 | 4*a* | 0.8596 | 0.4936 | 0.7370 |
| Li4 | 4*a* | 0.3306 | 0.3202 | 0.7205 |
| Li5 | 4*a* | 0.8192 | 0.1809 | 0.7411 |
| H1 | 4*a* | 0.1837 | 0.1548 | 0.8406 |
| H2 | 4*a* | 0.6476 | 0.3407 | 0.8464 |
| H3 | 4*a* | 0.8410 | 0.4614 | 0.0353 |
| H4 | 4*a* | 0.1893 | 0.4859 | 0.8438 |
| H5 | 4*a* | 0.3852 | 0.3124 | 0.0392 |
| H6 | 4*a* | 0.6969 | 0.0029 | 0.0952 |
| H7 | 4*a* | 0.5195 | 0.4813 | ­0.1920 |
| H8 | 4*a* | 0.1568 | 0.1586 | 0.0952 |
| H9 | 4*a* | 0.8173 | 0.1877 | –0.0256 |
| H10 | 4*a* | 0.5044 | 0.1606 | 0.7068 |
| H11 | 4*a* | 0.5107 | 0.1704 | 0.2053 |

**Table S2: Structural parameters of high-pressure phase of Li_5_MoH_11_ the most stable above the compression rate of 53.5 % (corresponding to the *Pc* structure in the main text). The lattice cell is an orthorhombic with space group *Pc* (No. 7) and lattice parameters are *a* = 8.758 Å, *b* = 3.960 Å, *c* = 8.205 Å and *β* = 114.397˚.**

| Atom | Wyckoff notation | *x* | *y* | *z* |
| --- | --- | --- | --- | --- |
| Mo1 | 2*a* | 0.2403 | 0.7675 | 0.1623 |
| Mo2 | 2*a* | 0.7339 | 0.7717 | 0.3835 |
| Li1 | 2*a* | 0.2572 | 0.7417 | 0.4646 |
| Li2 | 2*a* | 0.2084 | 0.7472 | 0.8576 |
| Li3 | 2*a* | –0.0590 | 0.7488 | –0.0456 |
| Li4 | 2*a* | ­–0.0089 | 0.7345 | 0.2805 |
| Li5 | 2*a* | 0.0326 | 0.7441 | 0.5720 |
| Li6 | 2*a* | 0.4879 | 0.7069 | 0.0507 |
| Li7 | 2*a* | 0.4845 | 0.7606 | 0.4271 |
| Li8 | 2*a* | 0.4885 | 0.7468 | 0.7838 |
| Li9 | 2*a* | 0.7065 | 0.7477 | 0.6655 |
| Li10 | 2*a* | 0.7475 | 0.7453 | 0.0911 |
| H1 | 2*a* | 0.1122 | 0.4877 | 0.4712 |
| H2 | 2*a* | 0.1085 | 0.0055 | 0.4679 |
| H3 | 2*a* | 0.1084 | 0.5255 | 0.2210 |
| H4 | 2*a* | 0.0969 | –0.0269 | 0.2193 |
| H5 | 2*a* | 0.3634 | 0.4933 | 0.1102 |
| H6 | 2*a* | 0.3609 | –0.0338 | 0.0728 |
| H7 | 2*a* | 0.3511 | 0.4942 | 0.3501 |
| H8 | 2*a* | 0.3484 | 0.0138 | 0.3541 |
| H9 | 2*a* | 0.4575 | 0.7878 | 0.2307 |
| H10 | 2*a* | 0.2554 | 0.8071 | 0.6652 |
| H11 | 2*a* | 0.7238 | 0.8085 | 0.8729 |
| H12 | 2*a* | 0.8729 | 0.5085 | 0.5664 |
| H13 | 2*a* | 0.8773 | –0.0085 | 0.5725 |
| H14 | 2*a* | –0.0591 | 0.6748 | 0.7439 |
| H15 | 2*a* | 0.8489 | 0.0279 | 0.7934 |
| H16 | 2*a* | 0.8431 | 0.5244 | 0.2992 |
| H17 | 2*a* | 0.6147 | 0.4977 | 0.4707 |
| H18 | 2*a* | 0.6125 | 0.0175 | 0.4726 |
| H19 | 2*a* | 0.6046 | 0.4920 | 0.7239 |
| H20 | 2*a* | 0.5966 | 0.0259 | 0.6998 |
| H21 | 2*a* | 0.0045 | 0.8196 | 0.7830 |
| H22 | 2*a* | 0.4773 | 0.7371 | 0.5936 |

**Table S3: Structural parameters of high-pressure phase [1] of Li_5_MoH_11_. The lattice cell is an orthorhombic with space group *Ama*2 (No. 40) and lattice parameters are *a* = 7.122 Å, *b* = 7.679 Å and *c* = 4.388 Å.**

| Atom | Wyckoff notation | *x* | *y* | *z* |
| --- | --- | --- | --- | --- |
| Mo1 | 4*b* | 1/4 | 0.0047 | –0.0018 |
| Li1 | 4*b* | 1/4 | 0.8254 | 0.5137 |
| Li2 | 4*b* | 1/4 | 0.6755 | –0.0313 |
| Li3 | 4*a* | 0 | 0 | 0.3553 |
| Li4 | 8*c* | 0.4930 | 0.6783 | 0.3126 |
| H1 | 8*c* | 0.6228 | 0.8360 | 0.1603 |
| H2 | 8*c* | 0.6258 | 0.1694 | 0.1117 |
| H3 | 4*b* | 1/4 | 0.4896 | 0.8669 |
| H4 | 8*c* | 0.6245 | 0.5276 | 0.1786 |
| H5 | 4*a* | 0 | 0 | 0.0003 |
| H6 | 4*b* | 1/4 | 0.1786 | 0.7900 |
| H7 | 8*c* | 0.4998 | 0.8325 | 0.4903 |

**Table S4: Structural parameters of high-pressure phase [2] of Li_5_MoH_11_. The lattice cell is a monoclinic with space group *P*2_1_/*m* (No. 11) and lattice parameters are *a* = 3.835 Å, *b* = 7.654 Å, *c* = 4.487 Å and *β* = 114.36˚.**

| Atom | Wyckoff notation | *x* | *y* | *z* |
| --- | --- | --- | --- | --- |
| Mo1 | 2*a* | 0 | 0 | 0 |
| Li1 | 4*f* | 0.2919 | 0.0218 | 0.5827 |
| Li2 | 2*e* | 0.4803 | 1/4 | 0.0180 |
| Li3 | 2*e* | 0.7479 | 1/4 | 0.5523 |
| Li4 | 2*e* | 0.1388 | 1/4 | 0.33337 |
| H1 | 4*f* | 0.0990 | 0.1330 | 0.7353 |
| H2 | 4*f* | 0.6253 | 0.1340 | 0.7749 |
| H3 | 4*f* | 0.6088 | 0.8816 | 0.7207 |
| H4 | 2*e* | –0.0372 | 1/4 | –0.0423 |
| H5 | 4*f* | 0.1464 | 0.8807 | 0.7318 |
| H6 | 2*c* | 1/2 | 0 | 0 |
| H7 | 2*e* | 0.3989 | 1/4 | 0.6621 |

**Table S5: Structural parameters of high-pressure phase [3] of Li_5_MoH_11_. The lattice cell is a monoclinic with space group *Pc* (No. 7) and lattice parameters are *a* = 7.802 Å, *b* = 3.493 Å, *c* = 7.296 Å and *β* = 115.05˚.**

| Atom | Wyckoff notation | *x* | *y* | *z* |
| --- | --- | --- | --- | --- |
| Mo1 | 2*a* | 0.2372 | 0.7507 | 0.1607 |
| Mo2 | 2*a* | 0.7315 | 0.7545 | 0.3861 |
| Li1 | 2*a* | 0.2492 | 0.7480 | 0.4573 |
| Li2 | 2*a* | 0.2089 | 07510 | 0.8595 |
| Li3 | 2*a* | –0.0589 | 0.7532 | –0.0446 |
| Li4 | 2*a* | –0.0127 | 0.7453 | 0.2786 |
| Li5 | 2*a* | 0.0310 | 0.7463 | 0.5682 |
| Li6 | 2*a* | 0.4835 | 0.7466 | 0.0441 |
| Li7 | 2*a* | 0.4806 | 0.7521 | 0.4330 |
| Li8 | 2*a* | 0.4830 | 0.7499 | 0.7845 |
| Li9 | 2*a* | 0.7160 | 0.7503 | 0.6670 |
| Li10 | 2*a* | 0.7463 | 0.7483 | 0.0948 |
| H1 | 2*a* | 0.1005 | 0.4991 | 0.4554 |
| H2 | 2*a* | 0.1012 | –0.0051 | 0.4577 |
| H3 | 2*a* | 0.0952 | 0.5163 | 0.2248 |
| H4 | 2*a* | 0.0892 | –0.0166 | 0.2183 |
| H5 | 2*a* | 0.3746 | 0.4894 | 0.0926 |
| H6 | 2*a* | 0.3743 | 0.0097 | 0.0900 |
| H7 | 2*a* | 0.3556 | 0.4716 | 0.3594 |
| H8 | 2*a* | 0.3555 | 0.0309 | 0.3590 |
| H9 | 2*a* | 0.4838 | 0.7485 | 0.2417 |
| H10 | 2*a* | 0.2517 | 0.7503 | 0.6630 |
| H11 | 2*a* | 0.7188 | 0.7544 | 0.8731 |
| H12 | 2*a* | 0.8833 | 0.4895 | 0.5786 |
| H13 | 2*a* | 0.8845 | 0.0064 | 0.5835 |
| H14 | 2*a* | –0.0668 | 0.6756 | 0.7328 |
| H15 | 2*a* | 0.8531 | 0.0155 | 0.7931 |
| H16 | 2*a* | 0.8529 | 0.5130 | 0.2984 |
| H17 | 2*a* | 0.6053 | 0.4768 | 0.4738 |
| H18 | 2*a* | 0.6051 | 0.0270 | 0.4742 |
| H19 | 2*a* | 0.5938 | 0.5069 | 0.6975 |
| H20 | 2*a* | 0.5921 | –0.0058 | 0.6943 |
| H21 | 2*a* | 0.0106 | 0.8194 | 0.7908 |
| H22 | 2*a* | 0.4895 | 0.7421 | 0.6053 |

**Table S6: Structural parameters of high-pressure phase [4] of Li_5_MoH_11_. The lattice cell is a monoclinic with space group *Pc* (No. 7) and lattice parameters are *a* = 7.801 Å, *b* = 3.492 Å, *c* = 7.296 Å and *β* = 115.05˚.**

| Atom | Wyckoff notation | *x* | *y* | *z* |
| --- | --- | --- | --- | --- |
| Mo1 | 2*a* | 0.0405 | 0.2493 | 0.8405 |
| Mo2 | 2*a* | 0.5461 | 0.2455 | 0.6152 |
| Li1 | 2*a* | 0.0284 | 0.2520 | 0.5439 |
| Li2 | 2*a* | 0.0688 | 0.2490 | 0.1417 |
| Li3 | 2*a* | 0.3366 | 0.2468 | 0.0458 |
| Li4 | 2*a* | 0.2904 | 0.2547 | 0.7226 |
| Li5 | 2*a* | 0.2467 | 0.2537 | 0.4330 |
| Li6 | 2*a* | 0.7941 | 0.2534 | –0.0428 |
| Li7 | 2*a* | 0.7970 | 0.2479 | 0.5682 |
| Li8 | 2*a* | 0.7947 | 0.2501 | 0.2168 |
| Li9 | 2*a* | 0.5617 | 0.2497 | 0.3343 |
| Li10 | 2*a* | 0.5314 | 0.2517 | –0.0936 |
| H1 | 2*a* | 0.1764 | 0.0051 | 0.5435 |
| H2 | 2*a* | 0.1772 | 0.5009 | 0.5459 |
| H3 | 2*a* | 0.1885 | 0.0166 | 0.7829 |
| H4 | 2*a* | 0.1824 | 0.4837 | 0.7765 |
| H5 | 2*a* | –0.0967 | –0.0097 | –0.0887 |
| H6 | 2*a* | –0.0969 | 0.5106 | –0.0914 |
| H7 | 2*a* | –0.0779 | –0.0309 | 0.6422 |
| H8 | 2*a* | –0.0780 | 0.5285 | 0.6419 |
| H9 | 2*a* | 0.7938 | 0.2515 | 0.7595 |
| H10 | 2*a* | 0.0260 | 0.2497 | 0.3382 |
| H11 | 2*a* | 0.5589 | 0.2456 | 0.1281 |
| H12 | 2*a* | 0.3931 | –0.0065 | 0.4177 |
| H13 | 2*a* | 0.3943 | 0.5105 | 0.4226 |
| H14 | 2*a* | 0.4246 | –0.0155 | 0.2082 |
| H15 | 2*a* | 0.3445 | 0.3244 | 0.2685 |
| H16 | 2*a* | 0.4248 | 0.4870 | 0.7029 |
| H17 | 2*a* | 0.6726 | –0.0270 | 0.5270 |
| H18 | 2*a* | 0.6724 | 0.5232 | 0.5275 |
| H19 | 2*a* | 0.6856 | 0.0058 | 0.3070 |
| H20 | 2*a* | 0.6838 | 0.4931 | 0.3037 |
| H21 | 2*a* | 0.2671 | 0.1806 | 0.2104 |
| H22 | 2*a* | 0.7881 | 0.2579 | 0.3959 |

**Table S7: Structural parameters of high-pressure phase [5] of Li_5_MoH_11_. The lattice cell is a monoclinic with space group *P*2_1_ (No. 4) and lattice parameters are *a* = 3.379 Å, *b* = 6.873 Å, *c* = 3.818 Å and *β* = 115.52˚.**

| Atom | Wyckoff notation | *x* | *y* | *z* |
| --- | --- | --- | --- | --- |
| Mo1 | 2*a* | 0.6371 | 0.7756 | 0.7715 |
| Li1 | 2*a* | 0.3336 | 0.7961 | 0.1807 |
| Li2 | 2*a* | –0.0786 | 0.7816 | 0.3538 |
| Li3 | 2*a* | 0.1488 | 0.0061 | 0.8025 |
| Li4 | 2*a* | 0.5771 | 0.0569 | 0.6677 |
| Li5 | 2*a* | 0.8471 | 0.0582 | 0.2216 |
| H1 | 2*a* | 0.7703 | 0.4215 | 0.5051 |
| H2 | 2*a* | 0.4651 | 0.1479 | –0.0746 |
| H3 | 2*a* | 0.5033 | –0.0309 | 0.2812 |
| H4 | 2*a* | 0.8581 | 0.2855 | 0.2286 |
| H5 | 2*a* | –0.0453 | 0.1449 | –0.0655 |
| H6 | 2*a* | 0.5424 | –0.0742 | 0.0252 |
| H7 | 2*a* | 0.7871 | –0.0740 | ­0.5132 |
| H8 | 2*a* | –0.0076 | –0.0805 | 0.0557 |
| H9 | 2*a* | 0.2780 | 0.0280 | 0.2105 |
| H10 | 2*a* | 0.2006 | 0.1334 | 0.5022 |
| H11 | 2*a* | 0.2029 | 0.6433 | 0.4803 |

**Table S8: Structural parameters of high-pressure phase [6] of Li_5_MoH_11_. The lattice cell is a monoclinic with space group *P*2_1_ (No. 4) and lattice parameters are *a* = 3.379 Å, *b* = 6.873 Å, *c* = 3.819 Å and *β* = 115.60˚.**

| Atom | Wyckoff notation | *x* | *y* | *z* |
| --- | --- | --- | --- | --- |
| Mo1 | 2*a* | 0.3621 | 0.7756 | 0.2285 |
| Li1 | 2*a* | 0.6662 | 0.7961 | 0.8192 |
| Li2 | 2*a* | 0.0785 | 0.7816 | 0.6461 |
| Li3 | 2*a* | 0.4228 | 0.0061 | 0.3326 |
| Li4 | 2*a* | 0.1530 | 0.0569 | 0.7783 |
| Li5 | 2*a* | 0.8501 | 0.0582 | 0.1969 |
| H1 | 2*a* | 0.1416 | 0.4215 | 0.7712 |
| H2 | 2*a* | 0.2295 | 0.1479 | 0.4947 |
| H3 | 2*a* | 0.7215 | –0.0309 | 0.7893 |
| H4 | 2*a* | 0.5345 | 0.2855 | 0.0745 |
| H5 | 2*a* | 0.4969 | 0.1449 | 0.7197 |
| H6 | 2*a* | 0.2126 | –0.0742 | 0.4862 |
| H7 | 2*a* | 0.0073 | –0.0740 | ­–0.0559 |
| H8 | 2*a* | 0.4567 | –0.0805 | –0.0264 |
| H9 | 2*a* | 0.0450 | 0.0280 | 0.0661 |
| H10 | 2*a* | 0.7993 | 0.1334 | 0.4974 |
| H11 | 2*a* | 0.7977 | 0.6433 | 0.5201 |

**Table S9: Structural parameters of high-pressure phase [7] of Li_5_MoH_11_. The lattice cell is a monoclinic with space group *Cc* (No. 9) and lattice parameters are *a* = 4.421 Å, *b* = 7.485 Å, *c* = 7.253 Å and *β* = 90.588˚.**

| Atom | Wyckoff notation | *x* | *y* | *z* |
| --- | --- | --- | --- | --- |
| Mo1 | 4*a* | –0.0001 | 0.0043 | –0.0257 |
| Li1 | 4*a* | 0.0249 | 0.3291 | –0.0427 |
| Li2 | 4*a* | 0.4784 | 0.1607 | –0.0459 |
| Li3 | 4*a* | 0.8473 | 0.4959 | 0.7316 |
| Li4 | 4*a* | 0.3175 | 0.3206 | 0.7178 |
| Li5 | 4*a* | 0.8094 | 0.1779 | 0.7342 |
| H1 | 4*a* | 0.1642 | 0.1610 | 0.8361 |
| H2 | 4*a* | 0.6377 | 0.3363 | 0.8443 |
| H3 | 4*a* | 0.8568 | 0.4788 | 0.0201 |
| H4 | 4*a* | 0.1806 | 0.4856 | 0.8414 |
| H5 | 4*a* | 0.5452 | 0.3124 | 0.0850 |
| H6 | 4*a* | 0.1830 | 4734 | 0.0914 |
| H7 | 4*a* | 0.5021 | 0.4983 | ­0.1985 |
| H8 | 4*a* | 0.1650 | 0.1687 | 0.0862 |
| H9 | 4*a* | 0.7973 | 0.1851 | –0.0085 |
| H10 | 4*a* | 0.4941 | 0.1620 | 0.7071 |
| H11 | 4*a* | 0.4967 | 0.1684 | 0.2100 |

**Table S10: Structural parameters of high-pressure phase [8] of Li_5_MoH_11_. The lattice cell is a monoclinic with space group *P*2_1_ (No. 4) and lattice parameters are *a* = 3.839 Å, *b* = 7.673 Å, *c* = 7.466 Å and *β* = 114.211˚.**

| Atom | Wyckoff notation | *x* | *y* | *z* |
| --- | --- | --- | --- | --- |
| Mo1 | 2*a* | –0.0128 | 0.0382 | 0.0042 |
| Li1 | 2*a* | 0.2868 | 0.0706 | 0.5901 |
| Li2 | 2*a* | 0.6999 | 0.0228 | 0.4217 |
| Li3 | 2*a* | 0.4802 | 0.2893 | 0.0179 |
| Li4 | 2*a* | 0.7333 | 0.2900 | 0.5551 |
| Li5 | 2*a* | 0.1280 | 0.2927 | 0.3251 |
| H1 | 2*a* | 0.1006 | 0.1779 | 0.7552 |
| H2 | 2*a* | 0.6317 | 0.1765 | 0.7866 |
| H3 | 2*a* | 0.6016 | –0.0747 | 0.7141 |
| H4 | 2*a* | 0.3813 | 0.1615 | 0.2790 |
| H5 | 2*a* | –0.0637 | –0.0834 | 0.3048 |
| H6 | 2*a* | 0.3809 | –0.0868 | 0.2157 |
| H7 | 2*a* | 0.0151 | 0.8176 | ­0.0481 |
| H8 | 2*a* | 0.1479 | –0.0717 | 0.7438 |
| H9 | 2*a* | 0.5389 | 0.0305 | 0.0108 |
| H10 | 2*a* | 0.8427 | 0.1663 | 0.2737 |
| H11 | 2*a* | 0.6317 | 0.8020 | 0.3442 |

**Table S11: Structural parameters of high-pressure phase [9] of Li_5_MoH_11_. The lattice cell is a triclinic with space group *P*1 (No. 1) and lattice parameters are *a* = 3.417 Å, *b* = 3.859 Å, *c* = 6.920 Å, *α* = 76.039˚, *β* = 76.804˚ and *γ* = 65.973˚.**

| Atom | Wyckoff notation | *x* | *y* | *z* |
| --- | --- | --- | --- | --- |
| Mo1 | 1*a* | 0.8266 | 0.1152 | –0.0427 |
| Mo2 | 1*a* | 0.6211 | –0.0126 | 0.4608 |
| Li1 | 1*a* | 0.5293 | 0.6965 | –0.0527 |
| Li2 | 1*a* | 0.1139 | 0.5466 | –0.0568 |
| Li3 | 1*a* | 0.0285 | –0.0423 | 0.6583 |
| Li4 | 1*a* | 0.7821 | 0.4588 | 0.6768 |
| Li5 | 1*a* | 0.3803 | 0.1837 | 0.7550 |
| Li6 | 1*a* | –0.0074 | 0.6012 | 0.2031 |
| Li7 | 1*a* | 0.6046 | 0.4138 | 0.1968 |
| Li8 | 1*a* | 0.2905 | 0.0446 | 0.1980 |
| Li9 | 1*a* | 0.3395 | 0.5598 | 0.4592 |
| Li10 | 1*a* | –0.0665 | 0.3953 | 0.4422 |
| H1 | 1*a* | 0.2408 | 0.8656 | 0.8189 |
| H2 | 1*a* | 0.4759 | 0.4509 | 0.8221 |
| H3 | 1*a* | 0.6682 | 0.7671 | 0.0914 |
| H4 | 1*a* | –0.0352 | 0.4625 | 0.8168 |
| H5 | 1*a* | 0.3725 | 0.3814 | 0.0831 |
| H6 | 1*a* | –0.0260 | 0.3283 | 0.0929 |
| H7 | 1*a* | 0.7585 | 0.0023 | 0.2073 |
| H8 | 1*a* | 0.1990 | 0.8156 | 0.0895 |
| H9 | 1*a* | 0.3294 | 0.1057 | –0.0309 |
| H10 | 1*a* | 0.1505 | –0.0264 | 0.4451 |
| H11 | 1*a* | 0.1837 | 0.2556 | 0.5914 |
| H12 | 1*a* | –0.0102 | 0.6718 | 0.5876 |
| H13 | 1*a* | 0.6584 | 0.2698 | 0.5879 |
| H14 | 1*a* | 0.5914 | 0.8275 | 0.7093 |
| H15 | 1*a* | 0.2946 | 0.3051 | 0.3124 |
| H16 | 1*a* | 0.4137 | 0.6809 | 0.6103 |
| H17 | 1*a* | 0.0621 | 0.7827 | 0.3285 |
| H18 | 1*a* | 0.5960 | 0.7092 | 0.3226 |
| H19 | 1*a* | 0.7620 | 0.8708 | 0.8187 |
| H20 | 1*a* | 0.8935 | 0.2432 | 0.3078 |
| H21 | 1*a* | 0.2116 | 0.5694 | 0.7026 |
| H22 | 1*a* | 0.3122 | 0.6938 | 0.2483 |

**Table S12: Structural parameters of high-pressure phase [10] of Li_5_MoH_11_. The lattice cell is a triclinic with space group *P*1 (No. 1) and lattice parameters are *a* = 3.492 Å, *b* = 4.041 Å, *c* = 7.286 Å, *α* = 86.540˚, *β* = 76.207˚ and *γ* = 64.469˚.**

| Atom | Wyckoff notation | *x* | *y* | *z* |
| --- | --- | --- | --- | --- |
| Mo1 | 1*a* | 0.0614 | 0.0843 | 0.0404 |
| Mo2 | 1*a* | 0.3285 | 0.0406 | 0.5461 |
| Li1 | 1*a* | 0.7777 | 0.6654 | 0.0285 |
| Li2 | 1*a* | 0.3343 | 0.5104 | 0.0683 |
| Li3 | 1*a* | –0.0328 | –0.0307 | 0.3366 |
| Li4 | 1*a* | 0.6967 | 0.5698 | 0.2904 |
| Li5 | 1*a* | 0.4465 | 0.1060 | 0.2466 |
| Li6 | 1*a* | 0.4312 | 0.6047 | 0.7940 |
| Li7 | 1*a* | 0.0394 | 0.3857 | 0.7969 |
| Li8 | 1*a* | 0.6892 | 0.0857 | 0.7946 |
| Li9 | 1*a* | 0.0358 | 0.6179 | 0.5617 |
| Li10 | 1*a* | 0.6367 | 0.4431 | 0.5313 |
| H1 | 1*a* | 0.8780 | 0.8111 | 0.1773 |
| H2 | 1*a* | 0.0893 | 0.3544 | 0.1828 |
| H3 | 1*a* | 0.5284 | 0.8127 | –0.0965 |
| H4 | 1*a* | 0.6222 | 0.3492 | 0.1872 |
| H5 | 1*a* | 0.2668 | 0.3597 | –0.0783 |
| H6 | 1*a* | 0.7042 | 0.3655 | –0.0780 |
| H7 | 1*a* | 0.2272 | –0.0002 | 0.7938 |
| H8 | 1*a* | 0.0135 | 0.8029 | –0.0969 |
| H9 | 1*a* | 0.5765 | 0.0744 | 0.0259 |
| H10 | 1*a* | 0.8320 | 0.0272 | 0.5588 |
| H11 | 1*a* | 0.5404 | 0.2827 | 0.3944 |
| H12 | 1*a* | 0.3081 | 0.7356 | 0.4241 |
| H13 | 1*a* | 0.0286 | 0.2736 | 0.3927 |
| H14 | 1*a* | 0.7764 | 0.7405 | 0.4256 |
| H15 | 1*a* | 0.3957 | 0.3413 | 0.6724 |
| H16 | 1*a* | 0.1116 | 0.5322 | 0.3445 |
| H17 | 1*a* | 0.1269 | 0.7963 | 0.6860 |
| H18 | 1*a* | 0.6387 | 0.8010 | 0.6834 |
| H19 | 1*a* | 0.3815 | 0.8137 | 0.1764 |
| H20 | 1*a* | 0.8439 | 0.3443 | 0.6724 |
| H21 | 1*a* | 0.2733 | 0.5716 | 0.2668 |
| H22 | 1*a* | 0.8539 | 0.7211 | 0.7882 |

**Table S13: Structural parameters of high-pressure phase [11] of Li_5_MoH_11_. The lattice cell is a monoclinic with space group *Cc* (No. 9) and lattice parameters are *a* = 6.022 Å, *b* = 4.023 Å, *c* = 8.081 Å and *β* = 113.151˚.**

| Atom | Wyckoff notation | *x* | *y* | *z* |
| --- | --- | --- | --- | --- |
| Mo1 | 4*a* | 0.7638 | 0.2278 | –0.0338 |
| Li1 | 4*a* | 0.0837 | 0.2664 | 0.8934 |
| Li2 | 4*a* | 0.4421 | 0.2643 | 0.0395 |
| Li3 | 4*a* | 0.5913 | 0.0530 | 0.6879 |
| Li4 | 4*a* | 0.4265 | 0.4408 | 0.7556 |
| Li5 | 4*a* | 0.2466 | 0.0171 | 0.7169 |
| H1 | 4*a* | 0.2789 | 0.1007 | 0.8836 |
| H2 | 4*a* | 0.2817 | 0.3658 | 0.8591 |
| H3 | 4*a* | 0.0009 | 0.4501 | –0.0041 |
| H4 | 4*a* | 0.4962 | 0.1095 | 0.8178 |
| H5 | 4*a* | 0.2450 | 0.3538 | 0.0813 |
| H6 | 4*a* | 0.7044 | 0.1910 | 0.1414 |
| H7 | 4*a* | 0.0184 | 0.1166 | ­0.1220 |
| H8 | 4*a* | 0.2604 | 0.1080 | 0.0659 |
| H9 | 4*a* | 0.5371 | 0.4661 | –0.0472 |
| H10 | 4*a* | 0.0324 | 0.2203 | 0.7183 |
| H11 | 4*a* | 0.8213 | 0.2193 | 0.7831 |

**Table S14: Structural parameters of high-pressure phase [12] of Li_5_MoH_11_. The lattice cell is a monoclinic with space group *P*21 (No. 4) and lattice parameters are *a* = 2.761 Å, *b* = 7.870 Å, *c* = 4.287 Å and *β* = 104.995˚.**

| Atom | Wyckoff notation | *x* | *y* | *z* |
| --- | --- | --- | --- | --- |
| Mo1 | 2*a* | 0.0071 | –0.0129 | 0.0098 |
| Li1 | 2*a* | 0.6670 | –0.0955 | 0.3610 |
| Li2 | 2*a* | 0.3196 | –0.0995 | 0.6036 |
| Li3 | 2*a* | 0.8805 | 0.7243 | 0.7449 |
| Li4 | 2*a* | 0.5535 | 0.7246 | 0.0822 |
| Li5 | 2*a* | 0.1748 | 0.6847 | 0.4120 |
| H1 | 2*a* | 0.4715 | 0.8758 | –0.0469 |
| H2 | 2*a* | 0.8713 | 0.8856 | 0.6819 |
| H3 | 2*a* | 0.6332 | 0.0347 | 0.5887 |
| H4 | 2*a* | 0.1118 | 0.8365 | 0.2706 |
| H5 | 2*a* | 0.5421 | 0.0859 | 0.1452 |
| H6 | 2*a* | 0.4290 | 0.0605 | 0.4280 |
| H7 | 2*a* | 0.2528 | 0.0685 | ­0.7415 |
| H8 | 2*a* | –0.0635 | 0.1887 | –0.0762 |
| H9 | 2*a* | –0.0228 | 0.0548 | 0.3768 |
| H10 | 2*a* | 0.3836 | 0.7369 | 0.7315 |
| H11 | 2*a* | 0.3183 | 0.2292 | 0.5792 |

**Table S15: Structural parameters of high-pressure phase [13] of Li_5_MoH_11_. The lattice cell is a monoclinic with space group *P*21 (No. 4) and lattice parameters are *a* = 3.527 Å, *b* = 6.861 Å, *c* = 4.095 Å and *β* = 114.750˚.**

| Atom | Wyckoff notation | *x* | *y* | *z* |
| --- | --- | --- | --- | --- |
| Mo1 | 2*a* | 0.8212 | –0.0025 | 0.6530 |
| Li1 | 2*a* | 0.5054 | –0.0500 | 0.0269 |
| Li2 | 2*a* | 0.2936 | 0.8095 | 0.5860 |
| Li3 | 2*a* | 0.5974 | 0.7280 | 0.1761 |
| Li4 | 2*a* | 0.0717 | 0.7780 | 0.1277 |
| Li5 | 2*a* | 0.8495 | 0.6207 | 0.7121 |
| H1 | 2*a* | 0.1606 | 0.8055 | 0.8462 |
| H2 | 2*a* | 0.6773 | 0.7951 | 0.8459 |
| H3 | 2*a* | –0.0510 | –0.0400 | 0.0813 |
| H4 | 2*a* | –0.0824 | –0.0903 | 0.3164 |
| H5 | 2*a* | 0.5204 | 0.1863 | 0.5545 |
| H6 | 2*a* | 0.0244 | 0.1859 | 0.5493 |
| H7 | 2*a* | 0.7406 | 0.1094 | ­–0.0056 |
| H8 | 2*a* | 0.2207 | 0.0990 | –0.0218 |
| H9 | 2*a* | 0.3179 | 0.0146 | 0.6524 |
| H10 | 2*a* | 0.3809 | 0.6207 | 0.7719 |
| H11 | 2*a* | 0.6071 | 0.4109 | 0.6842 |
